# Mandible exosomal ssc-mir-133b regulates tooth development in miniature swine via endogenous apoptosis

**DOI:** 10.1038/s41413-018-0028-5

**Published:** 2018-09-11

**Authors:** Ye Li, Xinxin Wang, Jiali Ren, Xiaoshan Wu, Guoqing Li, Zhipeng Fan, Chunmei Zhang, Ang Li, Songlin Wang

**Affiliations:** 1 Molecular Laboratory for Gene Therapy and Tooth Regeneration, Beijing Key Laboratory of Tooth Regeneration and Function Reconstruction, School of Stomatology, Capital Medical University, Beijing, China; 2 Key Laboratory of Shaanxi Province for Craniofacial Precision Medicine Research, College of Stomatology, Xi’an Jiaotong University, Xi’an, China; 3 Laboratory of Molecular Signaling and Stem Cell Therapy, Beijing Key Laboratory of Tooth Regeneration and Function Reconstruction, School of Stomatology, Capital Medical University, Beijing, China; 4 Department of Biochemistry and Molecular Biology, Capital Medical University School of Basic Medical Sciences, Beijing, China

## Abstract

Signal transduction between different organs is crucial in the normal development of the human body. As an important medium for signal communication, exosomes can transfer important information, such as microRNAs (miRNAs), from donors to receptors. MiRNAs are known to fine-tune a variety of biological processes, including maxillofacial development; however, the underlying mechanism remains largely unknown. In the present study, transient apoptosis was found to be due to the expression of a miniature swine maxillofacial-specific miRNA, ssc-mir-133b. Upregulation of ssc-mir-133b resulted in robust apoptosis in primary dental mesenchymal cells in the maxillofacial region. Cell leukemia myeloid 1 (Mcl-1) was verified as the functional target, which triggered further downstream activation of endogenous mitochondria-related apoptotic processes during tooth development. More importantly, mandible exosomes were responsible for the initial apoptosis signal. An animal study demonstrated that ectopic expression of ssc-mir-133b resulted in failed tooth formation after 12 weeks of subcutaneous transplantation in nude mice. The tooth germ developed abnormally without the indispensable exosomal signals from the mandible.

## Tooth development: special delivery from the mandible

The delivery of the small regulatory molecule microRNA-133b via extracellular vesicles released from the lower jaw is required for tooth formation in pigs and mice. Several microRNAs have been implicated in tooth development, but their precise roles are poorly understood. Songlin Wang at Capital Medical University, China, and colleagues found that microRNA-133b causes temporary cell death at sites of molar development by reducing the levels of the pro-survival protein myeloid cell leukemia-1. Moreover, they showed that microRNA-133b is delivered from the lower jaw in exosomes and that interrupting this signal prevents tooth development. These findings highlight the importance of cross-talk between jaw and tooth tissue for normal development and reveal a possible mechanism for the prevention and treatment of abnormal tooth formation.

## Introduction

Normally developed organs are the result of the accurate spatiotemporal expression of related genes and appropriate signals “talking” between donors and receptors.^1–4^ Maxillofacial development is a complex process because different tissues and organs are involved.^5^ Teeth and the mandible are functionally and locally related tissues in the maxillofacial region, as they are adjacent to one another and disruptions that affect the mandible also negatively affect dental patterning during development.^6–9^ Cross-talk between the teeth and mandible are crucially important for maintaining the normal development of both tissues.^10–13^

As a newly discovered player in tissue and organ cross-talk, exosomes play important roles in diverse biological processes, such as tissue growth, organ development, and body immune regulation.^14–17^ The basis of exosome signal transduction is the multiple signaling molecules contained therein, among which microRNAs (miRNAs) have attracted the most attention in recent years.^18,19^ Exosomes can transfer miRNA information from donor to recipient cells, regulating the biological functions of the recipient cells.^20,21^

MiRNAs are known to be involved in the regulation of many important biological processes, including maxillofacial development. However, only a few functional studies have revealed specific miRNA functions. MiR-214 was first found to inhibit tooth mineralization by fine-tuning Clu and Tgfb1 during tooth development.^22,23^ By targeting multiple channels, miR-34a regulates the differentiation of dental papilla cells through ALP downregulation.^24^ MiR-200c/141 could regulate ameloblast differentiation during tooth development.^25^ MiR-200a-3p converts mesenchymal cells to epithelial cells by interacting with Pitx2 and beta-catenin.^26^ MiR-135a was reported to influence tooth formation by regulating the BMP pathway.^27^ MiR-27 promotes odontoblast differentiation through the Wnt/beta-catenin signaling pathway.^28^ MiR-224 can coordinate enamel mineralization by regulating ion transporter expression in ameloblasts.^29^ MiR-96 and Tbx1 function in a regulatory loop in tooth development.^30^ However, the actions of specific miRNAs in regulating tooth development are still not fully understood.

Apoptosis is a crucial process during embryonic development and an important morphogenetic event in maxillofacial development. Dysregulation of apoptosis may lead to tooth agenesis and mandible deficiency.^31,32^ The B-cell lymphoma 2 (Bcl-2) family plays a critical role in apoptosis. In particular, cell leukemia myeloid 1 (Mcl-1), one of the most important anti-apoptotic members of this family, inhibits apoptosis by interacting with pro-apoptotic members.^33,34^ In early studies, Mcl-1 deletion resulted in a lethal phenotype during mouse embryogenesis.^35^ However, it is still unclear whether Mcl-1 contributes to the progression of maxillofacial development.

In our previous study, five candidate miRNAs were specifically expressed in the maxillofacial region in miniature swine.^36^ The current study revealed that the developing mandible sends messages to developing teeth through exosomes. Exosomal ssc-mir-133b and its target gene Mcl-1 are important regulators of normal tooth development. Dysfunction in mandible exosomal signal transduction may lead to tooth agenesis during tooth development. Additionally, to the best of our knowledge, this is the first time that specific miRNAs have been studied in a large-animal maxillofacial development model. Our study may reveal how tooth development is regulated by the mandible and may provide insights into the possible mechanisms for the prevention and treatment of maxillofacial deformities.

## Results

### Expression pattern of ssc-mir-133b during premolar development

**Fig. 1:**
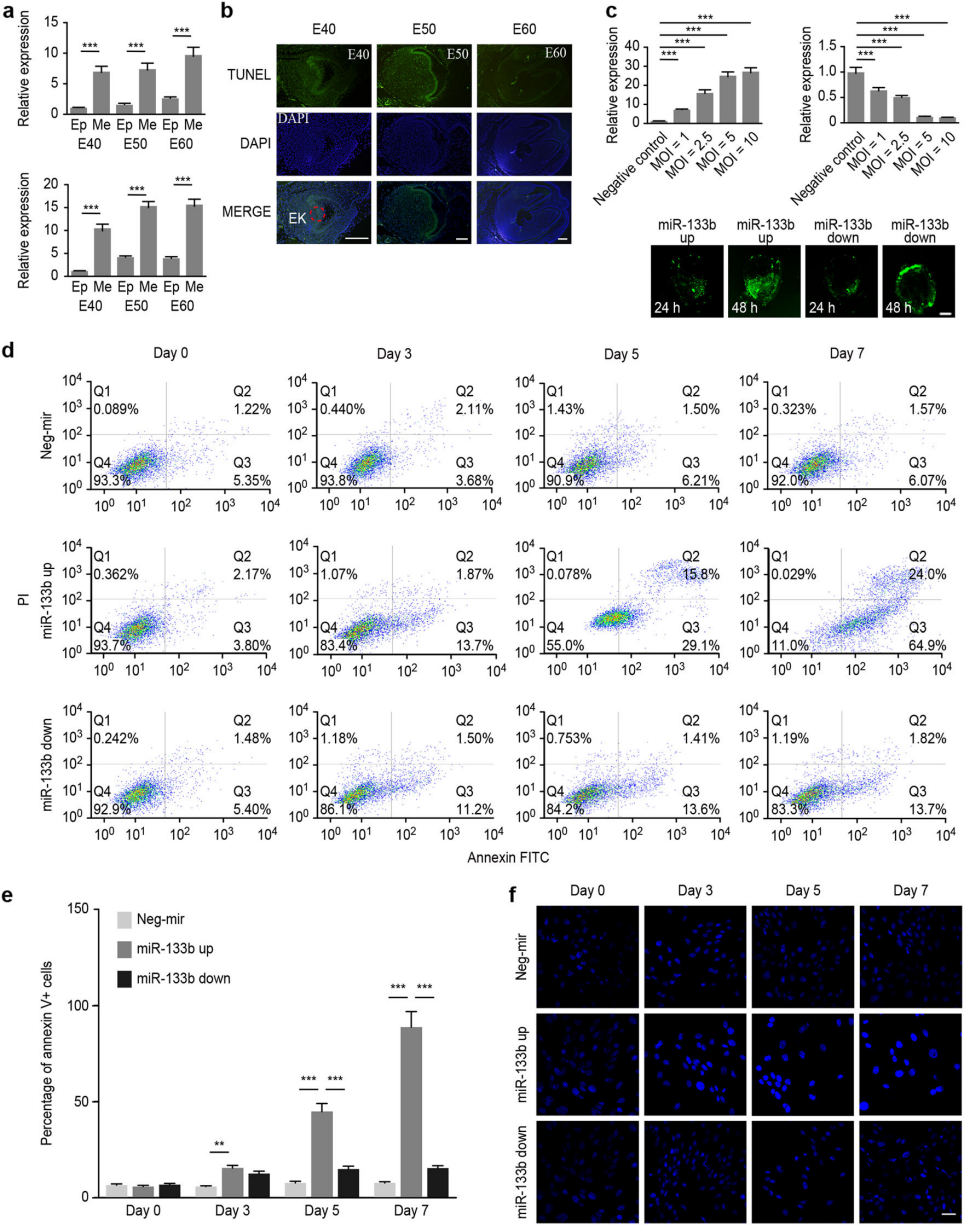
Ssc-mir-133b was highly related to cell apoptosis in the early stages of premolar development. **a** Relative expression of ssc-mir-133b in premolar dental epithelium and mesenchyme using qPCR. U6 was used as a control. Average ssc-mir-133b expression levels in premolar the dental mesenchyme were significantly higher than in the dental epithelium (upper panel). Similarly, the ssc-mir-133b expression levels in primary premolar dental mesenchyme cells were significantly higher than in dental epithelium cells (lower panel). ***P < 0.001. **b** Apoptosis was measured with a TUNEL assay in E40, E50, and E60 premolars. Scale bar = 200 μm. Enamel knots (EK, red dotted circle). **c** Relative ssc-mir-133b expression levels at 48 h after transfection of different MOIs of ssc-mir-133b lentiviral vectors and the efficiency of ssc-mir-133b lentiviral vector transfection. Average ssc-mir-133b expression levels after transfection with ssc-mir-133b overexpression lentiviral vector (upper left). average ssc-mir-133b expression levels after transfection with ssc-mir-133b inhibition lentiviral vector (upper right). ***P < 0.001. The efficiency of the ssc-mir-133b lentiviral vector (lower). **d** Cells dually stained with annexin-FITC/PI were investigated by flow cytometry analysis. Q1 quadrant represents the living cells; Q2 quadrant represents the early apoptotic cells; Q3 quadrant represents the late apoptotic cells; and Q4 quadrant represents the dead cells. **e** Statistical analysis of flow cytometry results. ***P < 0.001 and **P < 0.01. **f** Transfected premolar mesenchymal cells were stained with Hoechst 33342 on coverslips. A fluorescence microscope was used to photograph the results. Bar = 50 μm

In our previous study, we found that ssc-mir-133b was specifically expressed in premolars and was particularly located in the dental mesenchyme and enamel knots, the critical areas of tooth morphogenesis.^36,37^ To further validate its specific expression levels in the dental mesenchyme, we performed qPCR analysis. The results showed that ssc-mir-133b exhibited significantly higher expression in the dental mesenchyme than in the epithelium (Fig. 1a, upper panel). The analysis of primary cells from each tissue further confirmed the same expression patterns (Fig. 1a, lower panel).

### Ssc-mir-133b was strongly related to cell apoptosis in the early stages of premolar development

TUNEL assays were performed to evaluate apoptosis throughout the early stages of premolar development. Many apoptotic cells were located in the mesenchyme and enamel knots in the E40 and E50 premolars (Fig. 1b), which also showed expression of ssc-mir-133b. However, the apoptosis signal was almost invisible in the E60 premolar. Because a transient apoptotic signal was found in the ssc-mir-133b expression region, we hypothesized that the expression of ssc-mir-133b might be closely related to the apoptotic regulation of tooth development during this period. Additionally, since ssc-mir-133b was mainly expressed in premolar primary dental mesenchymal cells, these cells were selected to validate the role of ssc-mir-133b in the subsequent study. Lentivirus transfection was performed with neg-miR and miR-133b expression vectors and the most effective multiplicity of infection (MOI) value was determined by real-time PCR. For both the miR-133b overexpression and inhibition vectors, an MOI of 5 was chosen as the most effective transfection MOI (Fig. 1c, up). Moreover, the miR-133b expression vector was used to transfect tooth germs, and the effect of transfection is shown in Fig. 1c (bottom). Flow cytometry and Hoechst 33342 staining assays were performed in premolar primary dental mesenchymal cells. As shown in Fig. 1d, e, f, an increase in the number of apoptotic cells followed ssc-mir-133b overexpression.

### Ssc-mir-133b targeted Mcl-1 during premolar development

**Fig. 2:**
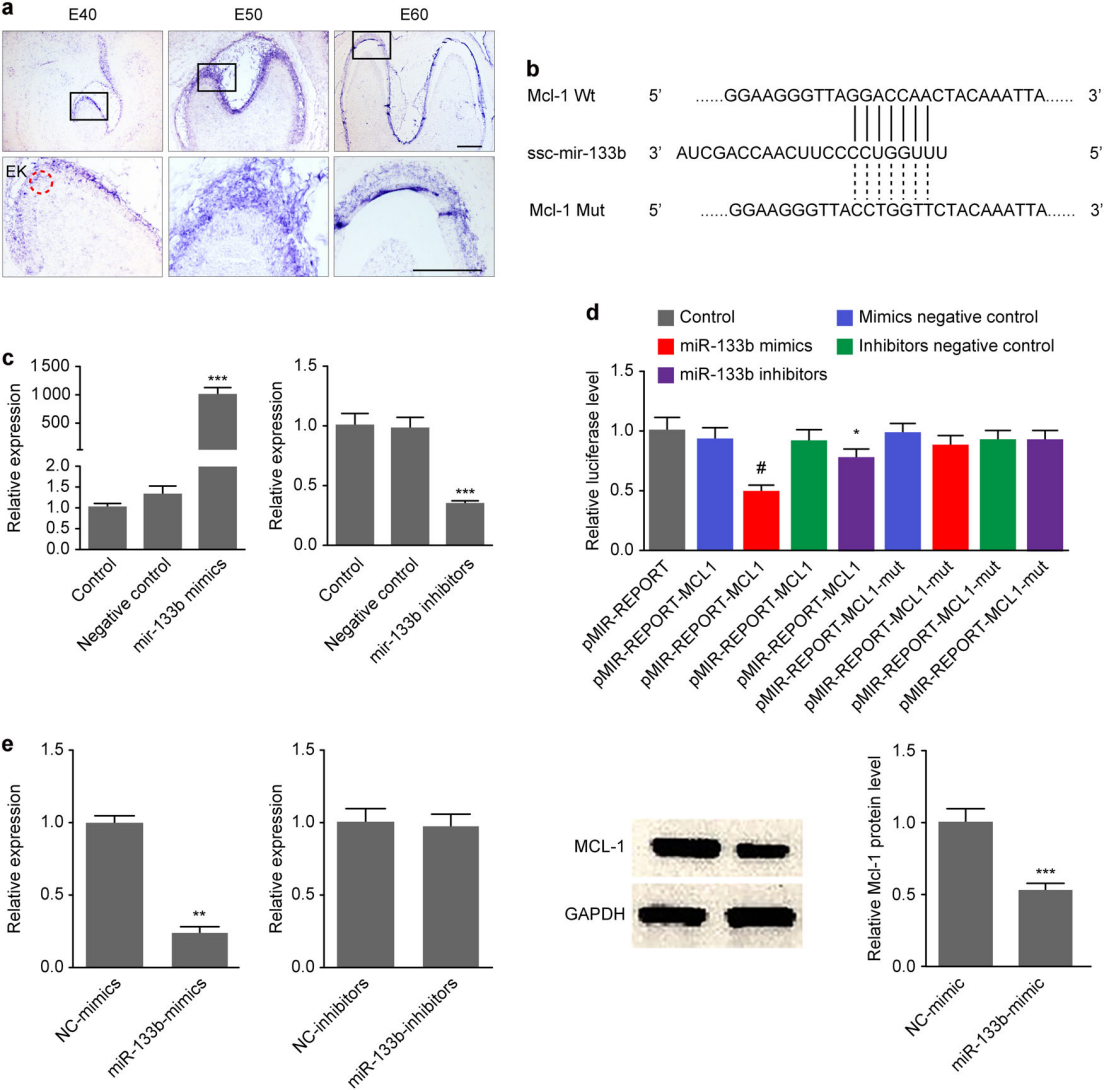
Mcl-1 is the downstream target gene of ssc-mir-133b during early premolar development. **a** In situ hybridization with DIG-labeled Mcl-1 cDNA in E40, E50, and E60 premolars. Scale bar = 200 μm. Enamel knots (EK, red dotted circle). **b** The Mcl-1 3′-UTR harbors one ssc-mir-133b-specific binding site. **c** Relative ssc-mir-133b expression levels at 48 h after transfection of ssc-mir-133b mimics and inhibitors. Relative ssc-mir-133b levels after transfection with ssc-mir-133b mimics (left). Relative ssc-mir-133b levels after transfection with ssc-mir-133b inhibitors (right). ***P < 0.001. **d** Relative luciferase activity is shown above. Reporter plasmids with the wild-type or mutant type Mcl-1 3’-UTR were co-transfected with ssc-mir-133b mimics or inhibitors and negative controls for both. ^#^*P* < 0.05 compared with pMIR-REPORT-MCL1 co-transfected with the ssc-mir-133b mimic negative control and *P < 0.05 compared with pMIR-REPORT-MCL1 co-transfected with ssc-mir-133b mimics. **e** qPCR for Mcl-1 mRNA levels after miR-133b mimic and inhibitor transfection (left) and Western blotting of Mcl-1 protein levels after miR-133b mimic transfection (right). **P < 0.01 and ***P < 0.001

Combined target gene prediction databases (TargetScan, PicTar, and miRBase) were searched for potential downstream target genes of ssc-mir-133b (Appendix Table 1). The bioinformatics data identified several apoptosis-related genes (such as Bcorl-1, Bcl2l-1, Bcl2l-2, Mcl-1, Bnop3l) as putative miR-133b target genes. However, when miR-133b was overexpressed in premolar primary dental mesenchymal cells, Mcl-1 was the only gene that was downregulated. Mcl-1 is a critical anti-apoptotic gene in Bcl-2-related apoptosis, and aberrant Mcl-1 expression may lead to abnormal apoptosis. Interestingly, in situ hybridization results in the present study showed that Mcl-1 had the same expression pattern as ssc-mir-133b in early tooth development, and more specifically in E40, E50, and E60 premolars (Fig. 2a). Therefore, Mcl-1 may be the ssc-mir-133b target gene that is involved in tooth development. As shown in Fig. 2b, Mcl-1 had a conserved ssc-mir-133b binding site in the structure of its 3′-untranslated region (UTR). To validate the regulatory role of ssc-mir-133b on Mcl-1, we cloned the wild-type Mcl-1 3′-UTR that contained the binding sites for ssc-mir-133b and a mutated version into the luciferase reporter plasmid pmir-glo (Promega, American). The cloned plasmids were co-transfected with ssc-mir-133b mimics and inhibitors separately in 293 T cells. The effects of the mimics and inhibitors on miR-133b are shown in Fig. 2c. In the wild-type Mcl-1 3′-UTR group, ssc-mir-133b suppressed approximately 50% of the luciferase activity, whereas luciferase activity was not affected in the mutant Mcl-1 3′-UTR group. Moreover, the ssc-mir-133b inhibitor substantially restored the luciferase activity of the wild-type Mcl-1 3′-UTR, whereas no significant change was found in the mutant Mcl-1 3′-UTR (Fig. 2d). As shown in Fig. 2e, ssc-mir-133b significantly downregulated Mcl-1 at both the mRNA and protein levels.

### Ssc-mir-133b mediated the endogenous mitochondria-related apoptotic process during premolar development

**Fig. 3:**
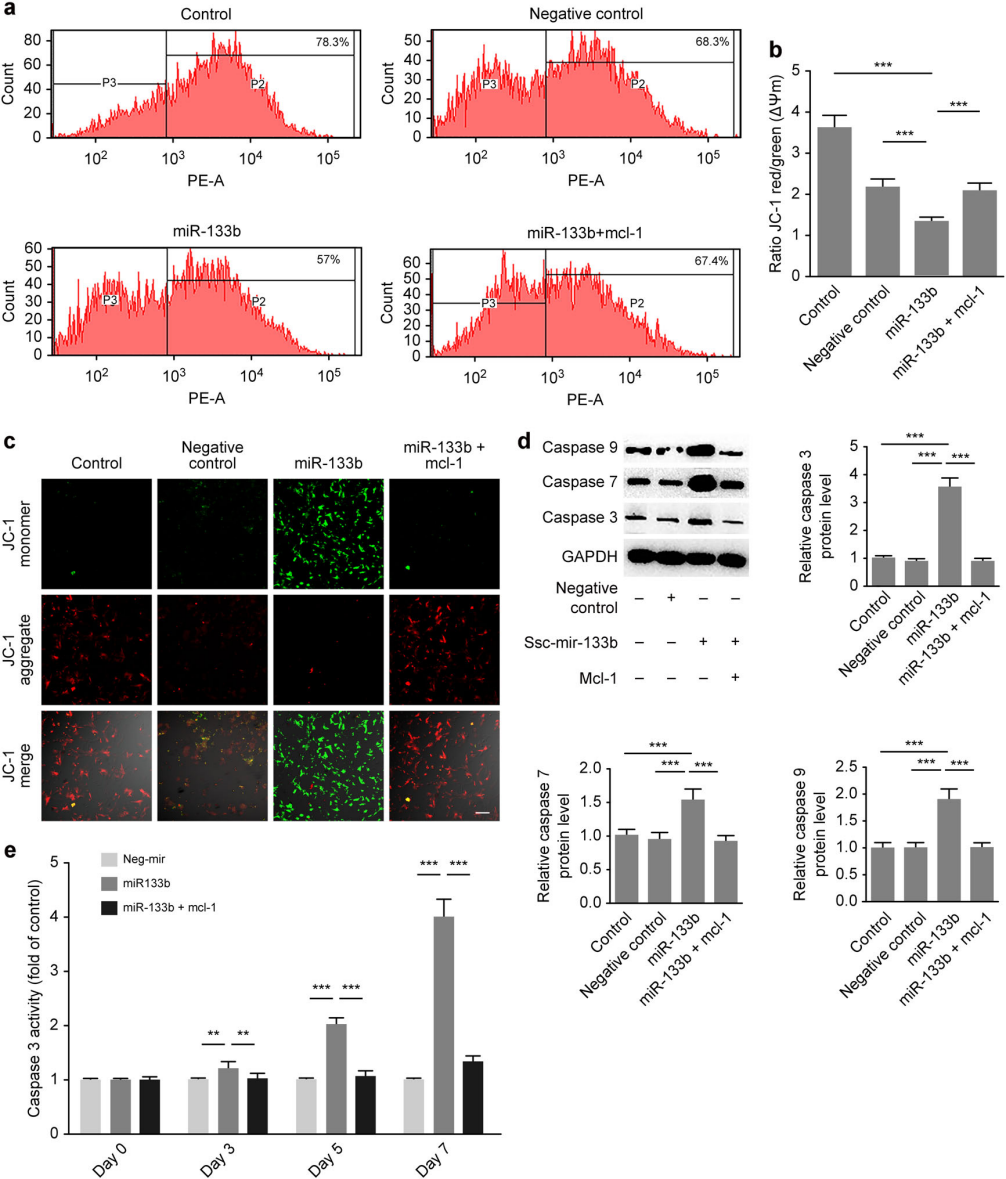
Ssc-mir-133b/Mcl-1 mediated endogenous mitochondria-related apoptotic processes in premolar mesenchymal cells. **a** Mitochondrial Δψm measured by the JC-1 probe. Distribution of JC-1 aggregates (PE channel) and monomers (FITC channel) was determined by flow cytometry. **b** Statistical analysis of the flow cytometry results. ***P < 0.001. **c** Representative images show JC-1 aggregates, JC-1 monomers and merged images of both. Increases in JC-1 monomers in cells are shown in cells transfected with miR-133b. Decreased JC-1 monomers in cells are shown in cells transfected with miR-133b along with Mcl-1. Scale bar = 50 μm. **d** Western blotting of endogenous mitochondria-related apoptotic markers after miR-133b transfection and Mcl-1 overexpression. ***P < 0.001. **e** Quantitative representation of caspase-3 activity in premolar mesenchymal cells. **P < 0.01 and ***P < 0.001

To further confirm that Mcl-1 is a downstream factor of ssc-mir-133b, we established a primary dental mesenchymal cell line stably overexpressing Mcl-1. Thus, the ssc-mir-133b/Mcl-1 downstream pathway was further investigated. Studies have shown that Mcl-1 is an important player in the endogenous mitochondrial apoptotic pathway.^38,39^ Therefore, ssc-mir-133b/Mcl-1 was hypothesized to be involved. The mitochondrial membrane potential (Δψm) is a valuable indicator of the apoptotic status of a cell. JC-1 has been widely used to measure Δψm. An increase in green fluorescence intensity indicates mitochondrial swelling and cell apoptosis. Figure 3a–c show representative profiles for JC-1 monomers and aggregates for dental mesenchymal cells, and the percentages of aggregates and monomers were quantified. Western blotting indicated that ectopic ssc-mir-133b promoted the expression of the endogenous apoptotic effectors caspase-3, caspase-7, and caspase-9, while Mcl-1 overexpression reversed this process (Fig. 3d). Next, we measured the activity of the apoptotic effector caspase-3. As expected, ssc-mir-133b significantly induced caspase-3 activity, whereas Mcl-1 rescued the endogenous mitochondria-related apoptotic process (Fig. 3e).

### The mandible regulated tooth germ development through the exosomal transfer of ssc-mir-133b

**Fig. 4:**
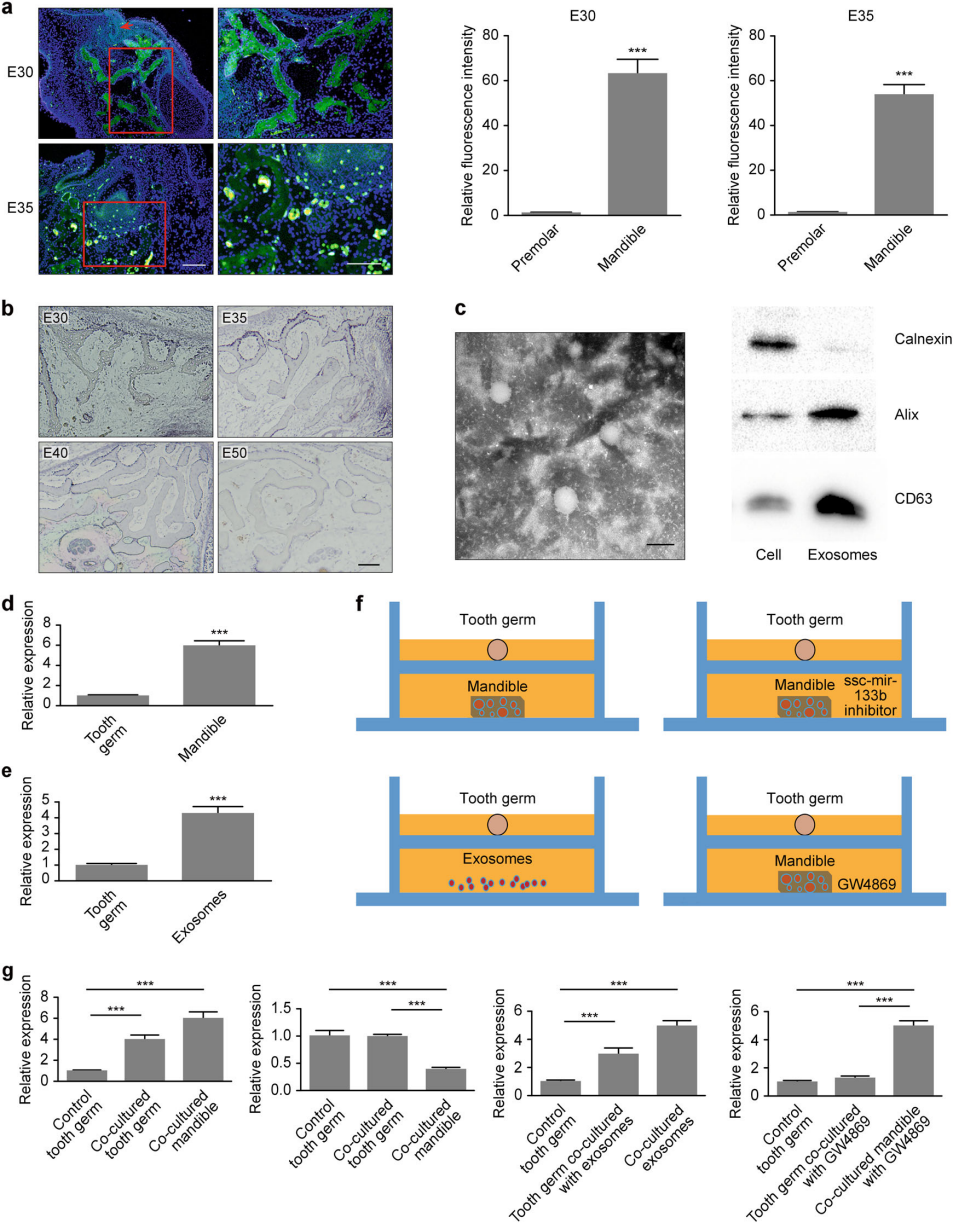
The mandible regulated tooth germ development through exosome-transferred ssc-mir-133b. **a** Apoptosis was measured with a TUNEL assay in E30 and E35 mandibles. Red arrowheads represent dental lamina. Scale bar = 200 μm. ***P < 0.001. **b** In situ hybridization of ssc-mir-133b in E30, E35, E40 and E50 mandibles. Scale bar = 200 μm. **c** Exosome characterization by transmission electron microscopy (**left**) and Western blotting (right). Scale bar = 50 nm. Calnexin is an endoplasmic reticulum marker and Alix and CD63 are exosome markers. **d** qPCR for ssc-mir-133b levels in E35 tooth germ and mandible. ***P < 0.001. **e** qPCR for ssc-mir-133b levels in E35 tooth germ and mandible exosomes. ***P < 0.001. **f** Transwell culture of tooth germ and mandible or mandible exosomes in vitro. **g** qPCR for ssc-mir-133b levels in tooth germ and mandible or mandible exosomes after Transwell culture. ***P < 0.001

Maxillofacial development is a complex process that involves signals in various tissues. Notably, in the present study, we found that in an early period of tooth development, such as on premolar day E30, no apoptotic signal was detected in tooth germs. More interestingly, significant signs of apoptosis were shown in the mandible during the corresponding period. By day E35 of embryonic development, weak apoptotic signals were detected in the dental mesenchyme (Fig. 4a). Additionally, strong ssc-mir-133b expression was found in the E30, E35, E40, and E50 mandibles (Fig. 4b). Therefore, we hypothesized that during tooth germ development, the mandible transmits apoptotic signals to the tooth germs and further regulates their development. To confirm this hypothesis, we separately isolated mandible and tooth germs from miniature swine at E35. Additionally, mandible exosomes were isolated and characterized (Fig. 4c). Mandibles showed robust ssc-mir-133b expression compared with the tooth germ, and exosomes from the mandible showed a similar ssc-mir-133b expression pattern compared with the tooth germ (Fig. 4d, e). As shown in Fig. 4f, g, when co-cultured with the mandible in a Transwell system, the tooth germ showed upregulated ssc-mir-133b expression. When ssc-mir-133b was inhibited in the mandible, co-culture of both tissues did not change the expression levels in the tooth germ. Exosomes were isolated after 72 h of mandible in vitro culture, and tooth germ ssc-mir-133b was upregulated when co-cultured with exosomes from the mandible. When an inhibitor of exosomes, GW4869, was added as a control, the expression of ssc-mir-133b in the tooth germ was not upregulated.

### Mandible-derived ssc-mir-133b is indispensable in normal tooth formation

**Fig. 5:**
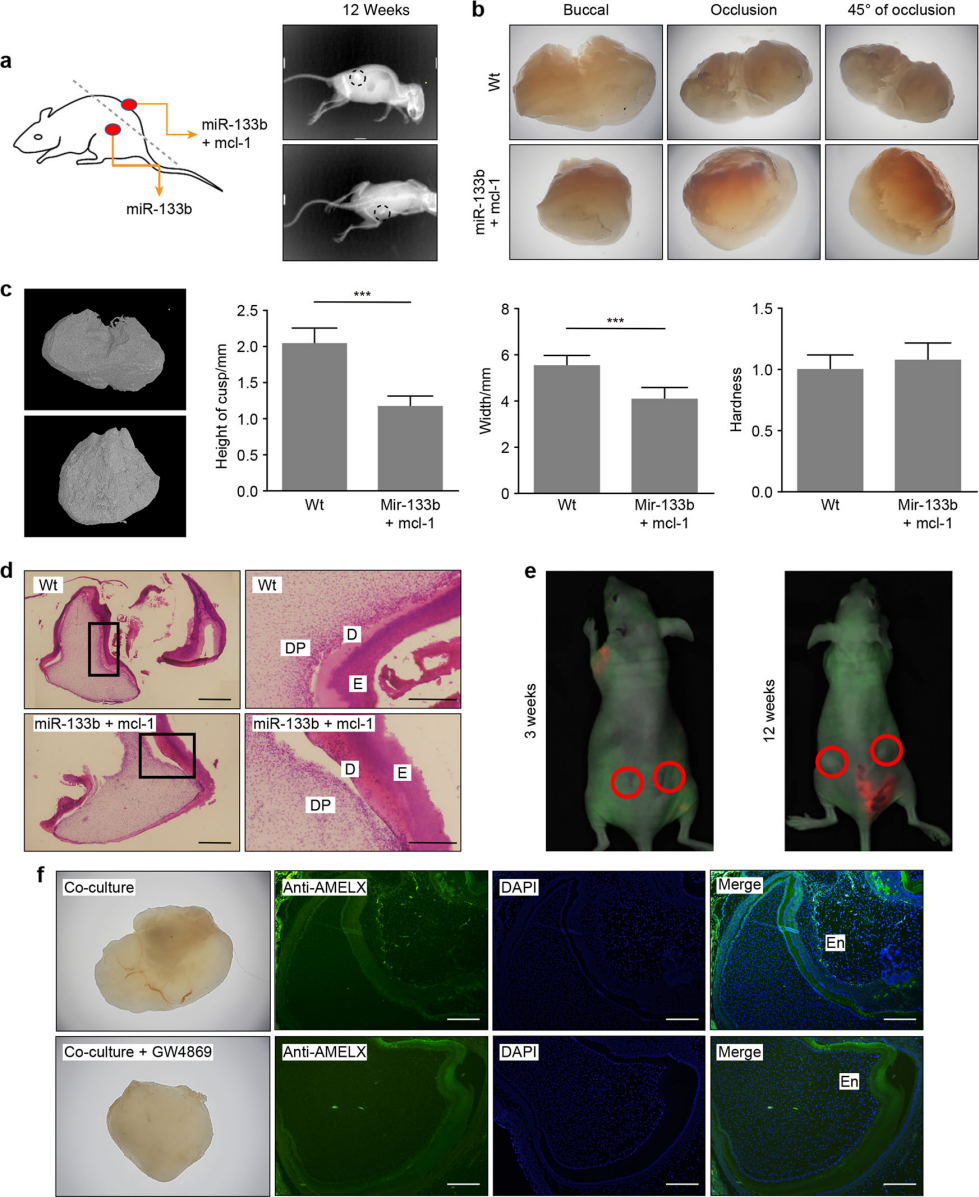
Mandible-derived ssc-mir-133b is indispensable in normal tooth formation. E40 premolars were cultured in vitro for 3 days with lentiviruses expressing neg-mir, miR-133b (left) and miR-133b + mcl-1 (right). They were then incubated subcutaneously in the back of nude mice for 12 weeks. **a** Tooth germs with lentiviruses expressing miR-133b failed to form, while tooth germs with lentiviruses expressing miR-133b + mcl-1 were readily observed 12 weeks after subcutaneous transplantation. **b** Morphological characteristics under a stereomicroscope. **c** 3D micro CT images of the negative control and Mcl-1 rescue groups. Tooth width, height of the teeth cups and tooth hardness were calculated for both groups. ***P < 0.001. **d** Haematoxylin and eosin staining of miR-133b + mcl-1 and neg-mir tooth germs. Scale bar = 200 μm. Dentin (**d**), dental pulp (DP), and enamel (**e**). **e** Status of tooth germ co-cultured with ma’ndible with (left side) and without GW4869 (right side). A red circle represents the transplantation area. **f** Morphology and amelogenin expression in tooth 12 weeks after co-culture with mandible with or without GW4869. Scale bar = 200 μm

We first investigated the effects of ssc-mir-133b/Mcl-1 on tooth formation. Subcutaneous transplantation and small animal in vivo imaging systems were used to evaluate the status of tooth formation in animal experiments (Fig. 5a). The results were collected 12 weeks after transplantation. Well-developed premolars were observed in the neg-mir group. However, no premolars were formed in the group with ssc-mir-133b overexpression (*N* = 6). As expected, developed premolars were detected in the Mcl-1 rescue group. A fully calcified premolar with two cusps was observed in the neg-mir group, while a calcified premolar with a smaller size and affected cusps was observed in the miR-133b + Mcl-1 group (Fig. 5b). Micro CT results showed that the morphological characteristics were influenced, though the tooth hardness remained the same in both the neg-mir and miR-133b + Mcl-1 groups (Fig. 5c). Haematoxylin and eosin (HE) staining of both the neg-mir and Mcl-1 overexpression groups is shown in Fig. 5d. Finally, the tooth germ and mandible were once again co-cultured in a Transwell system for 14 days, and then the tooth germs were subcutaneously transplanted into nude mice. The status of tooth development in nude mice was observed 3 and 12 weeks after transplantation (Fig. 5e). Amelogenin expression and morphological characteristics in both groups are shown in Fig. 5f. We found that mandibles without exosome signal transmission leads to dysfunctional tooth morphogenesis but normal enamel formation compared with normal mandible co-culture.

## Discussion

In our current study, we demonstrated that there is a transitory apoptotic process during tooth development that is regulated by the maxillofacial-specific ssc-mir-133b and its target gene Mcl-1. More importantly, the mandible is the origin of the initial signal and sends the apoptotic order to the tooth through exosomes to regulate normal tooth formation. We confirmed that mandible exosome-transmitted ssc-mir-133b can mediate the endogenous mitochondria-related apoptotic process during premolar development.

The specific spatiotemporal expression of particular genes is crucial for normal development.^40,41^ Therefore, the expression pattern of ssc-mir-133b in tooth germ was first investigated. Ssc-mir-133b was mainly expressed in the dental mesenchyme during the early stages of development, which indicated a regulatory role in the dental mesenchyme. Additionally, ssc-mir-133b expression was visible in the enamel knot, which is the center of tooth development,^42^ suggesting its crucial role in normal tooth development.

The present study demonstrated that ssc-mir-133b was highly related to apoptosis in the early stages of premolar development. During organ morphogenesis, apoptosis was reported to regulate cell number and tissue shape, but the specific mechanism is poorly understood.^43,44^ Apoptosis is a process in which specific cells are eliminated during the stages of development to maintain normal morphogenesis.^45^ Apparently, it is not a random event. From the present data, we hypothesized that ssc-mir-133b is spatiotemporally expressed during tooth morphogenesis as an effector to help remove unwanted cells in tooth germs to form a tooth with normal morphogenesis.

In the current study, Mcl-1 was confirmed as the downstream target of ssc-mir-133b that regulates tooth development during the early stages. During embryonic development, Mcl-1 is expressed in a wide variety of tissues.^34,46^ However, its role in tooth development has rarely been reported. Mcl-1 can be transcriptionally and translationally controlled by staurosporine and aspirin, as well as by non-coding RNAs, which revealed the diversity in its regulation.^47–49^ The co-localization of ssc-mir-133b and Mcl-1 together with the direct combination of both in the present research strongly suggested that ssc-mir-133b could directly regulate cell apoptosis through Mcl-1 during the early stage of tooth development, thus maintaining the normal tooth morphogenesis of miniature swine.

Mitochondria are organelles that coordinate caspase activation during apoptosis.^50^ The permeable outer mitochondrial membrane allows for cytochrome c release by integrating death signals from Bcl-2 family members, such as Mcl-1.^51^ Overexpression of ssc-mir-133b destroyed the mitochondrial membrane potential and upregulated the levels of caspase-3, caspace-7, and caspace-9 through the downregulation of Mcl-1, which suggested that ssc-mir-133b regulated tooth morphogenesis through endogenous mitochondria-related apoptotic processes.

In the current study, we observed that the mandible is the donor of ssc-mir-133b/Mcl-1 signaling in tooth germs during early development. More importantly, exosomes mediated this process. Recent studies have demonstrated that exosomes are predominantly endosomal in origin and contain a cargo of miRNAs, mRNAs, and proteins that are transferred from their original cells to target cells.^52^ Exosomes are released into the extracellular environment and interact with recipient cells via three pathways.^53^ They may enter cells via endocytic uptake or by direct fusion of the vesicles to the cell membrane. They may also transmit their contents through adhesion to the cell surface mediated by the interaction of a lipid–ligand receptor. These interactions indicate that exosomes possess pivotal roles in cell-to-cell communication in different physiological and pathological conditions. During development, cells communicate with one another to achieve coordinated tissue patterning.^54^ Signals are sent and received to regulate internal activities. In our case, exosomes from the mandible regulated the related process of tooth development through miRNAs. We isolated RNA from the mandible, mandible exosomes, and tooth germs to detect the expression of ssc-mir-133b and found that its expression in tooth germs was highly related to co-culture with mandible or mandible exosomes, which indicates that the regulation of tooth development is due to the cross-talk between the mandible and tooth germs. When exosomes were inhibited by GW4869, ssc-mir-133b/Mcl-1 regulation in the tooth germ was correspondingly inhibited.

In the present animal study, we found that overexpression of ssc-mir-133b in tooth germs could lead to failure of premolar formation. To date, little evidence has been found to indicate the key apoptotic molecules involved in tooth development. Msx-2, eda, and p21 have been reportedly correlated with cell death in odontogenesis.^55–57^ Mutations in these genes result in tooth agenesis. The loss of Mcl-1 expression leads to tooth formation failure, suggesting that Mcl-1 may be another candidate pathogenic gene related to tooth agenesis. Additionally, loss of exosome secretion leads to abnormal tooth formation during tooth germ development, indicating that exosomes are indispensable mediators in maxillofacial development.

**Fig. 6:**
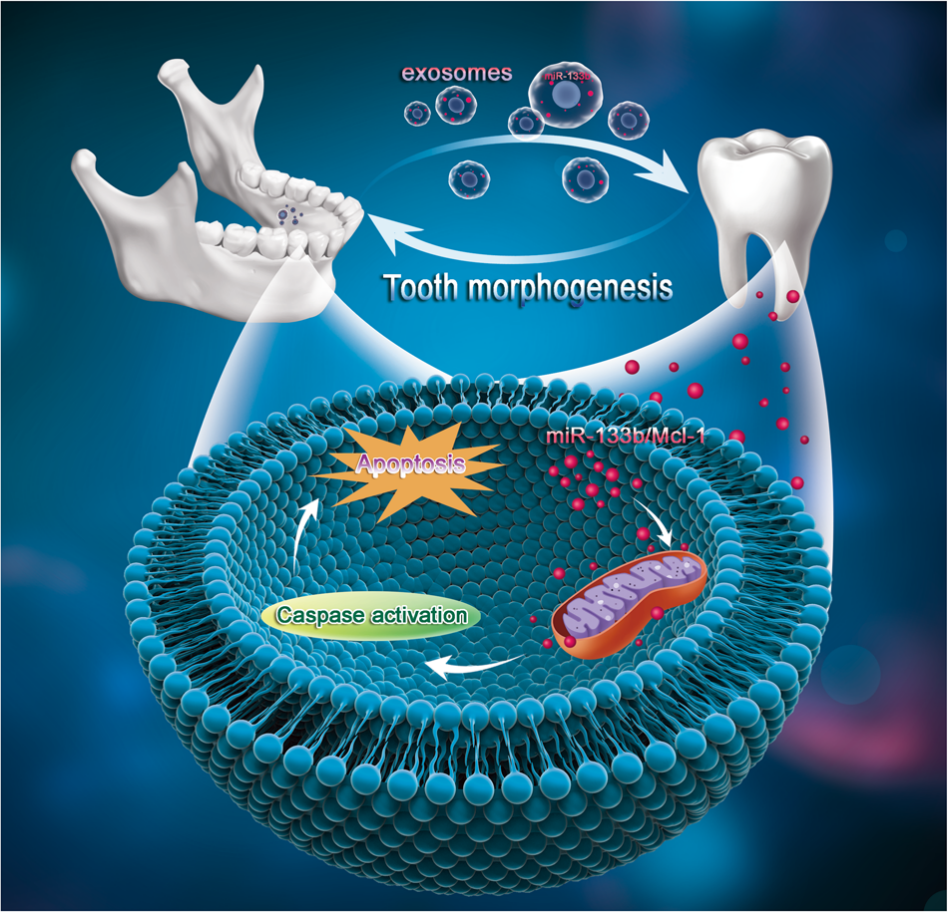
Schematic of mandible exosomal ssc-mir-133b regulation in tooth morphogenesis. In tooth development, the mandible secretes exosomes to transfer crucial regulatory ssc-mir-133b to tooth germs. Targeting Mcl-1 and related endogenous mitochondrial apoptotic signals regulates the morphology of the developing tooth

In conclusion, the existing data in the present study indicated that the mandible secretes exosomal ssc-mir-133b to the developing tooth to regulate its morphogenesis. Physiological ssc-mir-133b/Mcl-1 levels are critical for maintaining normal tooth development. By targeting Mcl-1, ssc-mir-133b activates apoptotic signals in developing dental mesenchyme, resulting in endogenous mitochondria-related apoptosis, which eventually regulates morphogenesis in the formation of premolars (Fig. 6).

## Materials and methods

### Animals

Large animals (Wuzhishan miniature swine) and adult nude male mice were purchased from the Laboratory Animal Company (Kexing and Weitong, Beijing, China). All related experiments were in compliance with protocols and policies approved (number CMU-B20100106) by the University Committee on Animals at Capital Medical University (Beijing, China).

### qPCR

Miniature swine premolars were harvested on embryonic days E40, E50, and E60, and the epithelium from each tooth was separated from the mesenchyme. Total RNA was extracted with the TRIzol reagent (TaKaRa, Japan). SYBR Green qPCR Mix (TaKaRa, Japan) was used to detect the expression of ssc-mir-133b in each sample; the primer sequences were consistent with those designed in a previous study.^36^ The final concentrations of reaction components for reverse transcription are as follows: 5 mmol·L^−1^ MgCl_2_; 1× reverse transcription buffer; 1 mmol·L^−1^ each dNTP; 1 U·μL^−1^ Recombinant RNasin® Ribonuclease Inhibitor; 15 U·μg^−1^ reverse transcriptase; 0.5 μg Oligo(dT); random primers per microgram RNA; and 50 ng·μL^−1^ total RNA. The reverse transcription parameters were as follows: 37 °C for 60 min and 85 °C for 5 s. For the qPCR assay, each reaction contained 10 μL Premix Ex Taq (2 × ), 0.4 μL PCR forward primer (10 μmol·L^−1^), 0.4 μL PCR reverse primer (10 μmol·L^−1^), 0.4 μL ROX Reference Dye II (50 × ), 2 μL cDNA template, and 6 μL sterile purified water. The PCR was performed with 40 cycles at 95 °C for 5 s and 60 °C for 34 s. All reactions were performed in triplicate.

### TUNEL staining

Mandibles with tooth germs were collected and fixed in 4% paraformaldehyde. The samples were then decalcified in 10% EDTA and treated by a series of increasing concentrations of ethanol for dehydration. Tissues were embedded and sliced. Each slide was deparaffinized and rehydrated. Then, 1 μg·mL proteinase K was used for digestion at 37 °C for 15 min. TUNEL reagents (Beyotime Biotechnology, Shanghai, China) were then applied according to the manufacturer’s instructions followed by DAPI staining.

### Tissue and cell culture

Premolar and mandibular tissues from miniature swine were maintained in a Transwell system with DMEM/F12 (1:1) medium supplemented with 20% FBS. Primary premolar mesenchymal cells were cultured in a 60-mm dish in DMEM/F12 (1:1) supplemented with 10% FBS. The tissues and cells were incubated at 37 °C with 5% CO_2_.

### Ssc-mir-133b lentiviral vector transduction

Tooth germs and cells were cultured for 3 days before lentiviral vector (1E + 8 TU·mL) transfection. Different MOI values (1, 2.5, 5, and 10) were used for overexpression and inhibition of ssc-mir-133b with the lentiviral vector (purchased from GeneChem Corporation, Shanghai, China) to determine the most appropriate MOI.

### Flow cytometry

Primary premolar mesenchymal cells that had been transfected with a lentiviral vector for 3, 5, or 7 days to overexpress or inhibit ssc-mir-133b expression were trypsinized and resuspended in PBS. After centrifugation, FITC-Annexin V and PI were added, and cells were incubated for 20 min in the dark at 4 °C. Finally, 500 µL of binding buffer was added to terminate the reaction.

### Hoechst 33342 staining

The cells were seeded at a density of 1 × 10^5^ per mL. After overnight attachment, the cells were transduced with a ssc-mir-133b overexpression or inhibition vector. After 3, 5, and 7 days, Hoechst 33342 (Sigma-Aldrich Corporation, St. Louis, MO, USA) fluorescence staining was used to detect the apoptotic morphological changes after lentiviral vector transfection.

### In situ hybridization

We examined the expression of the putative target Mcl-1 to determine whether it was co-localized with ssc-mir-133b in early tooth development. A full-length Mcl-1 cDNA clone was used as a template with a DIG label. Hybridization was conducted at 55 °C overnight. The expression of ssc-mir-133b in the mandible was also detected.

### Luciferase reporter assays

The miniature swine Mcl-1 mRNA 3′-UTR mutant and wild-type structures were inserted into pmir-glo (Promega) vectors. Then, 293 T cells were co-transfected with the vectors and ssc-mir-133b mimics (50 nmol·L^−1^) or inhibitors (100 nmol·L^−1^). Two days after transfection, a dual-luciferase reporter assay was performed to measure luciferase activity.

### Western blotting

Two days after transfection, RIPA lysis buffer containing protease inhibitors was used to extract the total protein. Total protein lysates were then centrifuged for 15 min at 12 000 × *g* at 4 °C. We then collected the supernatant for quantification using a BCA protein assay reagent kit (Beyotime). The total protein was denatured and then subjected to electrophoresis. Semi-dry electroblotting (1.5 mA·cm^−^^2^) was used to transfer the proteins to PVDF membranes. Primary antibodies were used for incubation overnight at 4 °C. Anti-caspase-9 (rabbit polyclonal), anti-caspase-7 (rabbit polyclonal), anti-caspase-3 (mouse mAb), and Mcl-1 (rabbit polyclonal) antibodies were obtained from Cell Signaling Technology. Secondary antibody incubation was performed for 2 h the next day followed by ECL exposure.

### Assessment of mitochondrial membrane potential (ψm)

Cells were incubated for 20 min in 2 μmol·L^−1^ JC-1 at 37 °C and resuspended in DMEM/F12, and flow cytometry was used to analyse and quantify the labeled cells.

### Caspase-3 activity assay

Primary premolar mesenchymal cells transfected with ssc-mir-133b and Mcl-1 were assessed in caspase-3 activity assays. Briefly, cells were resuspended in ice-cold cell lysis buffer for 15 min. After centrifugation at 10 000 r·min^−1^. for 3 min, the supernatant was collected for the assay. A plate reader was used to quantify caspase-3 activity at 405 nm.

### Exosome isolation and characterization

Mandibles were cultured with DMEM/F12 (1:1) medium supplemented with 20% exosome-depleted FBS for 72 h. Culture medium was collected for exosome isolation. ExoQuick TC was used according to the manufacturer’s instructions. Transmission electron microscopy was used to observe the appearance of the exosomes and Western blotting was used to detect specifically expressed proteins.

### Subcutaneous transplantation

Seven days after lentivirus transfection, tooth germs from E40 miniature swine were subcutaneously transplanted into the back of nude mice. Animal experiments were designed as follows: (1) a ssc-mir-133b-overexpressing tooth was implanted on the left side of nude mice and implanted on the right side in the Mcl-1 rescue group and (2) a neg-mir tooth was implanted on both sides of the nude mice. Tooth formation was examined 12 weeks later. For subcutaneous transplantation after Transwell culture, tooth germ co-cultured with mandible was implanted on the left side of the nude mice, while tooth germ co-cultured with GW4869-treated mandible was implanted on the right side of the nude mice. All animal experiments were in compliance with the protocols and policies approved by the University Committee on Animals at Capital Medical University.

### MicroCT (µCT) analysis

The tooth germs were scanned and reconstructed on a µCT. The width of the tooth and the height of tooth cups were rendered, and their mean intensity was calculated.

### Statistical analysis

The means and standard deviations were recorded and analyzed by ANOVA and the Student’s *t*-test. Between-group differences were tested using the Student’s unpaired *t*-test. Between-group and within-group differences were tested using repeated measure ANOVA. *P* < 0.05 was regarded as statistically significant.

### Data availability

The data that support the findings of this study are openly available.

## Electronic supplementary material

Appendix table 1
