# Supplementary material for: Mandible exosomal ssc-mir-133b regulates tooth development in miniature swine via endogenous apoptosis
Source: Bone Res. 2018 Sep 11;6:28. doi: 10.1038/s41413-018-0028-5 (PMC6131536; doi:10.1038/s41413-018-0028-5)
Supplement: Supplementary file 1 — Appendix table 1 [file 41413_2018_28_MOESM1_ESM.docx]

**Mandible exosomal ssc-mir-133b regulates tooth development of miniature swine via endogenous apoptosis**

Ye Li^1, 2^, Xinxin Wang^2^, Jiali Ren^2^, Xiaoshan Wu^1^, Guoqing Li^1^, Zhipeng Fan^3^, Chunmei Zhang^1^, Ang Li^1, 2*^, Songlin Wang^1, 4*^

**Appendix Table 1. Target gene prediction of ssc-mir-133b**

| \| Target gene \| Representative transcript \| Gene name \| \| --- \| --- \| --- \| \| LHFP \| ENST00000379589.3 \| lipoma HMGIC fusion partner \| \| SEC61B \| ENST00000498603.1 \| Sec61 beta subunit \| \| TAGLN2 \| ENST00000368096.1 \| transgelin 2 \| \| CETN3 \| ENST00000283122.3 \| centrin, EF-hand protein, 3 \| \| FTL \| ENST00000331825.6 \| ferritin, light polypeptide \| \| LDLRAP1 \| ENST00000374338.4 \| low density lipoprotein receptor adaptor protein 1 \| \| PTBP1 \| ENST00000350092.4 \| polypyrimidine tract binding protein 1 \| \| CLTA \| ENST00000433436.2 \| clathrin, light chain A \| \| TFAP2D \| ENST00000008391.3 \| transcription factor AP-2 delta (activating enhancer binding protein 2 delta) \| \| CELF4 \| ENST00000334919.5 \| CUGBP, Elav-like family member 4 \| \| SLC50A1 \| ENST00000368404.4 \| solute carrier family 50 (sugar efflux transporter), member 1 \| \| SYT2 \| ENST00000367267.1 \| synaptotagmin II \| \| TMEM200B \| ENST00000521452.1 \| transmembrane protein 200B \| \| TIMM17A \| ENST00000367287.4 \| translocase of inner mitochondrial membrane 17 homolog A (yeast) \| \| BICC1 \| ENST00000373886.3 \| bicaudal C homolog 1 (Drosophila) \| \| RBMX \| ENST00000570135.1 \| RNA binding motif protein, X-linked \| \| VKORC1 \| ENST00000300851.6 \| vitamin K epoxide reductase complex, subunit 1 \| \| ZNF354A \| ENST00000335815.2 \| zinc finger protein 354A \| \| SAMD5 \| ENST00000367474.1 \| sterile alpha motif domain containing 5 \| \| PRRT2 \| ENST00000300797.6 \| proline-rich transmembrane protein 2 \| \| LASP1 \| ENST00000318008.6 \| LIM and SH3 protein 1 \| \| ZIC3 \| ENST00000287538.5 \| Zic family member 3 \| \| PPP2CA \| ENST00000481195.1 \| protein phosphatase 2, catalytic subunit, alpha isozyme \| \| PAX7 \| ENST00000420770.2 \| paired box 7 \| \| C12orf43 \| ENST00000536407.2 \| chromosome 12 open reading frame 43 \| \| PPP2CB \| ENST00000221138.4 \| protein phosphatase 2, catalytic subunit, beta isozyme \| \| SLC30A7 \| ENST00000370112.4 \| solute carrier family 30 (zinc transporter), member 7 \| \| SGMS2 \| ENST00000394684.4 \| sphingomyelin synthase 2 \| \| GDNF \| ENST00000326524.2 \| glial cell derived neurotrophic factor \| \| SUMO1 \| ENST00000392246.2 \| small ubiquitin-like modifier 1 \| \| FOSL2 \| ENST00000379619.1 \| FOS-like antigen 2 \| \| SIMC1 \| ENST00000443967.1 \| SUMO-interacting motifs containing 1 \| \| SMARCD1 \| ENST00000394963.4 \| SWI/SNF related, matrix associated, actin dependent regulator of chromatin, subfamily d, member 1 \| \| GPM6A \| ENST00000280187.7 \| glycoprotein M6A \| \| DOLPP1 \| ENST00000540102.1 \| dolichyldiphosphatase 1 \| \| PTPRZ1 \| ENST00000393386.2 \| protein tyrosine phosphatase, receptor-type, Z polypeptide 1 \| \| TMEM167A \| ENST00000502346.1 \| transmembrane protein 167A \| \| CMPK1 \| ENST00000371873.5 \| cytidine monophosphate (UMP-CMP) kinase 1, cytosolic \| \| ARPP21 \| ENST00000428373.1 \| cAMP-regulated phosphoprotein, 21kDa \| \| PPP2R2D \| ENST00000422256.2 \| protein phosphatase 2, regulatory subunit B, delta \| \| LHX9 \| ENST00000367390.3 \| LIM homeobox 9 \| \| SLC6A1 \| ENST00000287766.4 \| solute carrier family 6 (neurotransmitter transporter), member 1 \| \| CNN2 \| ENST00000263097.4 \| calponin 2 \| \| KIRREL \| ENST00000368172.1 \| kin of IRRE like (Drosophila) \| \| XXYLT1 \| ENST00000310380.6 \| xyloside xylosyltransferase 1 \| \| LANCL2 \| ENST00000254770.2 \| LanC lantibiotic synthetase component C-like 2 (bacterial) \| \| TPM4 \| ENST00000300933.4 \| tropomyosin 4 \| \| TMOD3 \| ENST00000308580.7 \| tropomodulin 3 (ubiquitous) \| \| VPS54 \| ENST00000409558.4 \| vacuolar protein sorting 54 homolog (S. cerevisiae) \| \| COL25A1 \| ENST00000399132.1 \| collagen, type XXV, alpha 1 \| \| AGRP \| ENST00000290953.2 \| agouti related protein homolog (mouse) \| \| SGPP1 \| ENST00000247225.6 \| sphingosine-1-phosphate phosphatase 1 \| \| FAM57A \| ENST00000308278.8 \| family with sequence similarity 57, member A \| \| FOXL2 \| ENST00000330315.3 \| forkhead box L2 \| \| CAPN15 \| ENST00000219611.2 \| calpain 15 \| \| ZNF131 \| ENST00000505606.2 \| zinc finger protein 131 \| \| UBA2 \| ENST00000246548.4 \| ubiquitin-like modifier activating enzyme 2 \| \| KIF3C \| ENST00000264712.3 \| kinesin family member 3C \| \| EMP2 \| ENST00000359543.3 \| epithelial membrane protein 2 \| \| EIF4A1 \| ENST00000582746.1 \| eukaryotic translation initiation factor 4A1 \| \| AFTPH \| ENST00000238856.4 \| aftiphilin \| \| NRIP3 \| ENST00000309166.3 \| nuclear receptor interacting protein 3 \| \| AL117190.3 \| ENST00000599197.1 \| Esophagus cancer-related gene-2 interaction susceptibility protein; Uncharacterized protein \| \| GABARAPL1 \| ENST00000266458.5 \| GABA(A) receptor-associated protein like 1 \| \| B3GALNT1 \| ENST00000320474.4 \| beta-1,3-N-acetylgalactosaminyltransferase 1 (globoside blood group) \| \| GARNL3 \| ENST00000373387.4 \| GTPase activating Rap/RanGAP domain-like 3 \| \| RAP2C \| ENST00000342983.2 \| RAP2C, member of RAS oncogene family \| \| TRIM44 \| ENST00000299413.5 \| tripartite motif containing 44 \| \| GABPB2 \| ENST00000368918.3 \| GA binding protein transcription factor, beta subunit 2 \| \| ELFN1 \| ENST00000424383.2 \| extracellular leucine-rich repeat and fibronectin type III domain containing 1 \| \| TXLNA \| ENST00000373610.3 \| taxilin alpha \| \| STOM \| ENST00000286713.2 \| stomatin \| \| NAGS \| ENST00000293404.3 \| N-acetylglutamate synthase \| \| ANKRD28 \| ENST00000399451.2 \| ankyrin repeat domain 28 \| \| SLC6A6 \| ENST00000454876.2 \| solute carrier family 6 (neurotransmitter transporter), member 6 \| \| CRTAM \| ENST00000227348.4 \| cytotoxic and regulatory T cell molecule \| \| ZC3H11A \| ENST00000332127.4 \| zinc finger CCCH-type containing 11A \| \| PLEKHA3 \| ENST00000234453.5 \| pleckstrin homology domain containing, family A (phosphoinositide binding specific) member 3 \| \| SFXN2 \| ENST00000369893.5 \| sideroflexin 2 \| \| DCLRE1A \| ENST00000361384.2 \| DNA cross-link repair 1A \| \| PFAS \| ENST00000314666.6 \| phosphoribosylformylglycinamidine synthase \| \| KIAA1429 \| ENST00000437199.1 \| KIAA1429 \| \| QKI \| ENST00000392127.2 \| QKI, KH domain containing, RNA binding \| \| MAEA \| ENST00000303400.4 \| macrophage erythroblast attacher \| \| TBPL1 \| ENST00000237264.4 \| TBP-like 1 \| \| SF3B4 \| ENST00000271628.8 \| splicing factor 3b, subunit 4, 49kDa \| \| TMEM170B \| ENST00000379426.1 \| transmembrane protein 170B \| \| SOX4 \| ENST00000244745.1 \| SRY (sex determining region Y)-box 4 \| \| TSPAN18 \| ENST00000340160.3 \| tetraspanin 18 \| \| GCH1 \| ENST00000491895.2 \| GTP cyclohydrolase 1 \| \| RBMXL1 \| ENST00000321792.5 \| RNA binding motif protein, X-linked-like 1 \| \| DCBLD1 \| ENST00000338728.5 \| discoidin, CUB and LCCL domain containing 1 \| \| SH3GL2 \| ENST00000380607.4 \| SH3-domain GRB2-like 2 \| \| ZNF280C \| ENST00000370978.4 \| zinc finger protein 280C \| \| SLC39A1 \| ENST00000356205.4 \| solute carrier family 39 (zinc transporter), member 1 \| \| METTL21B \| ENST00000333012.5 \| methyltransferase like 21B \| \| FOXC1 \| ENST00000380874.2 \| forkhead box C1 \| \| PTPRK \| ENST00000368226.4 \| protein tyrosine phosphatase, receptor type, K \| \| WASF2 \| ENST00000536657.1 \| WAS protein family, member 2 \| \| RAVER1 \| ENST00000293677.6 \| ribonucleoprotein, PTB-binding 1 \| \| TRAM2 \| ENST00000182527.3 \| translocation associated membrane protein 2 \| \| VAMP2 \| ENST00000316509.6 \| vesicle-associated membrane protein 2 (synaptobrevin 2) \| \| ZNF710 \| ENST00000268154.4 \| zinc finger protein 710 \| \| SEPHS2 \| ENST00000542752.1 \| selenophosphate synthetase 2 \| \| EDEM1 \| ENST00000256497.4 \| ER degradation enhancer, mannosidase alpha-like 1 \| \| TTPAL \| ENST00000262605.4 \| tocopherol (alpha) transfer protein-like \| \| SOBP \| ENST00000317357.5 \| sine oculis binding protein homolog (Drosophila) \| \| BTBD3 \| ENST00000254977.3 \| BTB (POZ) domain containing 3 \| \| PDE8B \| ENST00000264917.5 \| phosphodiesterase 8B \| \| FAM46A \| ENST00000369754.3 \| family with sequence similarity 46, member A \| \| MAP3K2 \| ENST00000409947.1 \| mitogen-activated protein kinase kinase kinase 2 \| \| TM9SF3 \| ENST00000371142.4 \| transmembrane 9 superfamily member 3 \| \| RARB \| ENST00000437042.2 \| retinoic acid receptor, beta \| \| SPRN \| ENST00000414069.2 \| shadow of prion protein homolog (zebrafish) \| \| FOXQ1 \| ENST00000296839.2 \| forkhead box Q1 \| \| ZC3H7A \| ENST00000396516.2 \| zinc finger CCCH-type containing 7A \| \| COL8A1 \| ENST00000261037.3 \| collagen, type VIII, alpha 1 \| \| JDP2 \| ENST00000435893.2 \| Jun dimerization protein 2 \| \| POU6F2 \| ENST00000518318.2 \| POU class 6 homeobox 2 \| \| SNX15 \| ENST00000377244.3 \| sorting nexin 15 \| \| SNRK \| ENST00000429705.2 \| SNF related kinase \| \| RAD51L3-RFFL \| ENST00000593039.1 \| Uncharacterized protein \| \| MAML1 \| ENST00000292599.3 \| mastermind-like 1 (Drosophila) \| \| CELF6 \| ENST00000287202.5 \| CUGBP, Elav-like family member 6 \| \| LHX5 \| ENST00000261731.3 \| LIM homeobox 5 \| \| HS2ST1 \| ENST00000370550.5 \| heparan sulfate 2-O-sulfotransferase 1 \| \| SGK1 \| ENST00000367858.5 \| serum/glucocorticoid regulated kinase 1 \| \| ATP6AP2 \| ENST00000378438.4 \| ATPase, H+ transporting, lysosomal accessory protein 2 \| \| ELF3 \| ENST00000367284.5 \| E74-like factor 3 (ets domain transcription factor, epithelial-specific ) \| \| NUP160 \| ENST00000378460.2 \| nucleoporin 160kDa \| \| ANKRD46 \| ENST00000335659.3 \| ankyrin repeat domain 46 \| \| CERS2 \| ENST00000368954.5 \| ceramide synthase 2 \| \| ENPP5 \| ENST00000371383.2 \| ectonucleotide pyrophosphatase/phosphodiesterase 5 (putative) \| \| TUBB1 \| ENST00000217133.1 \| tubulin, beta 1 class VI \| \| CECR6 \| ENST00000399875.1 \| cat eye syndrome chromosome region, candidate 6 \| \| GLRA2 \| ENST00000218075.4 \| glycine receptor, alpha 2 \| \| PDE1C \| ENST00000396193.1 \| phosphodiesterase 1C, calmodulin-dependent 70kDa \| \| SV2A \| ENST00000369146.3 \| synaptic vesicle glycoprotein 2A \| \| DUSP1 \| ENST00000239223.3 \| dual specificity phosphatase 1 \| \| HAPLN1 \| ENST00000274341.4 \| hyaluronan and proteoglycan link protein 1 \| \| THRAP3 \| ENST00000354618.5 \| thyroid hormone receptor associated protein 3 \| \| VAT1 \| ENST00000355653.3 \| vesicle amine transport 1 \| \| NDRG1 \| ENST00000323851.7 \| N-myc downstream regulated 1 \| \| SYT1 \| ENST00000457153.2 \| synaptotagmin I \| \| FGF1 \| ENST00000360966.5 \| fibroblast growth factor 1 (acidic) \| \| SP3 \| ENST00000310015.6 \| Sp3 transcription factor \| \| CRK \| ENST00000398970.5 \| v-crk avian sarcoma virus CT10 oncogene homolog \| \| YPEL2 \| ENST00000312655.4 \| yippee-like 2 (Drosophila) \| \| ANKRD44 \| ENST00000282272.8 \| ankyrin repeat domain 44 \| \| MECOM \| ENST00000460814.1 \| MDS1 and EVI1 complex locus \| \| MED12L \| ENST00000474524.1 \| mediator complex subunit 12-like \| \| RIMS1 \| ENST00000348717.5 \| regulating synaptic membrane exocytosis 1 \| \| SCN2B \| ENST00000278947.5 \| sodium channel, voltage-gated, type II, beta subunit \| \| ARFIP2 \| ENST00000254584.2 \| ADP-ribosylation factor interacting protein 2 \| \| BCORL1 \| ENST00000540052.1 \| BCL6 corepressor-like 1 \| \| ATOX1 \| ENST00000521264.1 \| antioxidant 1 copper chaperone \| \| RFFL \| ENST00000315249.7 \| ring finger and FYVE-like domain containing E3 ubiquitin protein ligase \| \| SACM1L \| ENST00000389061.5 \| SAC1 suppressor of actin mutations 1-like (yeast) \| \| PFN2 \| ENST00000239940.7 \| profilin 2 \| \| CLCN6 \| ENST00000312413.6 \| chloride channel, voltage-sensitive 6 \| \| FGFR1 \| ENST00000397091.5 \| fibroblast growth factor receptor 1 \| \| UBE2Q1 \| ENST00000292211.4 \| ubiquitin-conjugating enzyme E2Q family member 1 \| \| BTBD10 \| ENST00000278174.5 \| BTB (POZ) domain containing 10 \| \| CCDC176 \| ENST00000394009.3 \| coiled-coil domain containing 176 \| \| PLCL2 \| ENST00000418129.2 \| phospholipase C-like 2 \| \| FOXP4 \| ENST00000373063.3 \| forkhead box P4 \| \| GZF1 \| ENST00000338121.5 \| GDNF-inducible zinc finger protein 1 \| \| XPO4 \| ENST00000400602.2 \| exportin 4 \| \| SNX30 \| ENST00000374232.3 \| sorting nexin family member 30 \| \| CORO1C \| ENST00000261401.3 \| coronin, actin binding protein, 1C \| \| LOXL4 \| ENST00000260702.3 \| lysyl oxidase-like 4 \| \| RPL17-C18orf32 \| ENST00000584895.1 \| RPL17-C18orf32 readthrough \| \| RB1CC1 \| ENST00000025008.5 \| RB1-inducible coiled-coil 1 \| \| EXD2 \| ENST00000409018.3 \| exonuclease 3'-5' domain containing 2 \| \| CPNE3 \| ENST00000198765.4 \| copine III \| \| SHISA5 \| ENST00000296444.2 \| shisa family member 5 \| \| YES1 \| ENST00000577961.1 \| v-yes-1 Yamaguchi sarcoma viral oncogene homolog 1 \| \| GXYLT1 \| ENST00000398675.3 \| glucoside xylosyltransferase 1 \| \| FBXL2 \| ENST00000484457.1 \| F-box and leucine-rich repeat protein 2 \| \| SYNRG \| ENST00000339208.6 \| synergin, gamma \| \| ARHGAP12 \| ENST00000311380.4 \| Rho GTPase activating protein 12 \| \| RBM23 \| ENST00000555209.1 \| RNA binding motif protein 23 \| \| VEGFC \| ENST00000280193.2 \| vascular endothelial growth factor C \| \| TNFRSF10B \| ENST00000276431.4 \| tumor necrosis factor receptor superfamily, member 10b \| \| CAP1 \| ENST00000372797.3 \| CAP, adenylate cyclase-associated protein 1 (yeast) \| \| KCTD15 \| ENST00000284006.6 \| potassium channel tetramerization domain containing 15 \| \| CTBP2 \| ENST00000337195.5 \| C-terminal binding protein 2 \| \| RAPH1 \| ENST00000319170.5 \| Ras association (RalGDS/AF-6) and pleckstrin homology domains 1 \| \| MEIS2 \| ENST00000397624.3 \| Meis homeobox 2 \| \| FAM117B \| ENST00000392238.2 \| family with sequence similarity 117, member B \| \| NAA40 \| ENST00000377793.4 \| N(alpha)-acetyltransferase 40, NatD catalytic subunit \| \| SLMO2 \| ENST00000355937.4 \| slowmo homolog 2 (Drosophila) \| \| CDK13 \| ENST00000181839.4 \| cyclin-dependent kinase 13 \| \| ENPEP \| ENST00000265162.5 \| glutamyl aminopeptidase (aminopeptidase A) \| \| PIKFYVE \| ENST00000264380.4 \| phosphoinositide kinase, FYVE finger containing \| \| SOGA3 \| ENST00000556132.1 \| SOGA family member 3 \| \| LAMB3 \| ENST00000356082.4 \| laminin, beta 3 \| \| LPIN2 \| ENST00000261596.4 \| lipin 2 \| \| CEP85L \| ENST00000368491.3 \| centrosomal protein 85kDa-like \| \| AKAP9 \| ENST00000356239.3 \| A kinase (PRKA) anchor protein 9 \| \| PPFIA2 \| ENST00000549396.1 \| protein tyrosine phosphatase, receptor type, f polypeptide (PTPRF), interacting protein (liprin), alpha 2 \| \| CELF1 \| ENST00000395290.2 \| CUGBP, Elav-like family member 1 \| \| KIAA1045 \| ENST00000242315.3 \| KIAA1045 \| \| SOCS2 \| ENST00000548537.1 \| suppressor of cytokine signaling 2 \| \| ETF1 \| ENST00000499810.2 \| eukaryotic translation termination factor 1 \| \| ELAVL1 \| ENST00000407627.2 \| ELAV like RNA binding protein 1 \| \| SGTB \| ENST00000381007.4 \| small glutamine-rich tetratricopeptide repeat (TPR)-containing, beta \| \| RCE1 \| ENST00000309657.3 \| RCE1 homolog, prenyl protein protease (S. cerevisiae) \| \| RBPJ \| ENST00000342320.4 \| recombination signal binding protein for immunoglobulin kappa J region \| \| BCL2L2 \| ENST00000250405.5 \| BCL2-like 2 \| \| DAPK2 \| ENST00000261891.3 \| death-associated protein kinase 2 \| \| PTBP2 \| ENST00000609116.1 \| polypyrimidine tract binding protein 2 \| \| KIAA1432 \| ENST00000414202.2 \| KIAA1432 \| \| RFC1 \| ENST00000349703.2 \| replication factor C (activator 1) 1, 145kDa \| \| PLEKHA8 \| ENST00000449726.1 \| pleckstrin homology domain containing, family A (phosphoinositide binding specific) member 8 \| \| TGFB2 \| ENST00000366930.4 \| transforming growth factor, beta 2 \| \| CMTM6 \| ENST00000205636.3 \| CKLF-like MARVEL transmembrane domain containing 6 \| \| UBXN7 \| ENST00000296328.4 \| UBX domain protein 7 \| \| PTPN22 \| ENST00000460620.1 \| protein tyrosine phosphatase, non-receptor type 22 (lymphoid) \| \| FBXW11 \| ENST00000296933.6 \| F-box and WD repeat domain containing 11 \| \| PAOX \| ENST00000368539.4 \| polyamine oxidase (exo-N4-amino) \| \| LRRC7 \| ENST00000310961.5 \| leucine rich repeat containing 7 \| \| ZNF740 \| ENST00000416904.3 \| zinc finger protein 740 \| \| KCNA6 \| ENST00000433855.1 \| potassium voltage-gated channel, shaker-related subfamily, member 6 \| \| MSN \| ENST00000360270.5 \| moesin \| \| ARPC5 \| ENST00000359856.6 \| actin related protein 2/3 complex, subunit 5, 16kDa \| \| DSN1 \| ENST00000426836.1 \| DSN1, MIS12 kinetochore complex component \| \| SLC25A36 \| ENST00000446041.2 \| solute carrier family 25 (pyrimidine nucleotide carrier ), member 36 \| \| ARL3 \| ENST00000260746.5 \| ADP-ribosylation factor-like 3 \| \| PAN3 \| ENST00000282391.5 \| PAN3 poly(A) specific ribonuclease subunit homolog (S. cerevisiae) \| \| PFKFB3 \| ENST00000379789.4 \| 6-phosphofructo-2-kinase/fructose-2,6-biphosphatase 3 \| \| XXbac-BPG32J3.20 \| ENST00000461287.1 \| \| \| MYRF \| ENST00000278836.5 \| myelin regulatory factor \| \| STX5 \| ENST00000377897.4 \| syntaxin 5 \| \| RAB30 \| ENST00000533486.1 \| RAB30, member RAS oncogene family \| \| CTGF \| ENST00000367976.3 \| connective tissue growth factor \| \| PTPRD \| ENST00000381196.4 \| protein tyrosine phosphatase, receptor type, D \| \| LDOC1 \| ENST00000370526.2 \| leucine zipper, down-regulated in cancer 1 \| \| PRDM6 \| ENST00000407847.4 \| PR domain containing 6 \| \| PPFIA3 \| ENST00000334186.4 \| protein tyrosine phosphatase, receptor type, f polypeptide (PTPRF), interacting protein (liprin), alpha 3 \| \| C20orf194 \| ENST00000453730.2 \| chromosome 20 open reading frame 194 \| \| RBMS1 \| ENST00000348849.3 \| RNA binding motif, single stranded interacting protein 1 \| \| KCND3 \| ENST00000369697.1 \| potassium voltage-gated channel, Shal-related subfamily, member 3 \| \| TFG \| ENST00000240851.4 \| TRK-fused gene \| \| CCDC117 \| ENST00000249064.4 \| coiled-coil domain containing 117 \| \| SUPT16H \| ENST00000216297.2 \| suppressor of Ty 16 homolog (S. cerevisiae) \| \| ARHGAP9 \| ENST00000424809.2 \| Rho GTPase activating protein 9 \| \| CTSV \| ENST00000259470.5 \| cathepsin V \| \| KCTD20 \| ENST00000373731.2 \| potassium channel tetramerization domain containing 20 \| \| MLLT3 \| ENST00000380338.4 \| myeloid/lymphoid or mixed-lineage leukemia (trithorax homolog, Drosophila); translocated to, 3 \| \| PRDM1 \| ENST00000369089.3 \| PR domain containing 1, with ZNF domain \| \| MEIS1 \| ENST00000444274.2 \| Meis homeobox 1 \| \| PRPF38A \| ENST00000257181.9 \| pre-mRNA processing factor 38A \| \| PITPNM2 \| ENST00000280562.5 \| phosphatidylinositol transfer protein, membrane-associated 2 \| \| TEAD1 \| ENST00000361905.4 \| TEA domain family member 1 (SV40 transcriptional enhancer factor) \| \| RUNX1T1 \| ENST00000523629.1 \| runt-related transcription factor 1; translocated to, 1 (cyclin D-related) \| \| PRKAB1 \| ENST00000229328.5 \| protein kinase, AMP-activated, beta 1 non-catalytic subunit \| \| PAPD5 \| ENST00000357464.3 \| PAP associated domain containing 5 \| \| TCF7 \| ENST00000518915.1 \| transcription factor 7 (T-cell specific, HMG-box) \| \| AOX1 \| ENST00000374700.2 \| aldehyde oxidase 1 \| \| ELF2 \| ENST00000394235.2 \| E74-like factor 2 (ets domain transcription factor) \| \| GRM5 \| ENST00000418177.2 \| glutamate receptor, metabotropic 5 \| \| EPHA7 \| ENST00000369303.4 \| EPH receptor A7 \| \| STXBP6 \| ENST00000396700.1 \| syntaxin binding protein 6 (amisyn) \| \| CASC3 \| ENST00000264645.7 \| cancer susceptibility candidate 3 \| \| DCP1A \| ENST00000607628.1 \| decapping mRNA 1A \| \| VAPB \| ENST00000475243.1 \| VAMP (vesicle-associated membrane protein)-associated protein B and C \| \| THNSL1 \| ENST00000524413.1 \| threonine synthase-like 1 (S. cerevisiae) \| \| ST8SIA3 \| ENST00000324000.3 \| ST8 alpha-N-acetyl-neuraminide alpha-2,8-sialyltransferase 3 \| \| AP1AR \| ENST00000274000.5 \| adaptor-related protein complex 1 associated regulatory protein \| \| TMEM57 \| ENST00000399766.3 \| transmembrane protein 57 \| \| TOR2A \| ENST00000458505.3 \| torsin family 2, member A \| \| PEX5L \| ENST00000467460.1 \| peroxisomal biogenesis factor 5-like \| \| SLC35F5 \| ENST00000245680.2 \| solute carrier family 35, member F5 \| \| PITPNB \| ENST00000335272.5 \| phosphatidylinositol transfer protein, beta \| \| GMEB1 \| ENST00000373816.1 \| glucocorticoid modulatory element binding protein 1 \| \| GRID2 \| ENST00000282020.4 \| glutamate receptor, ionotropic, delta 2 \| \| PREX1 \| ENST00000396220.1 \| phosphatidylinositol-3,4,5-trisphosphate-dependent Rac exchange factor 1 \| \| SVOP \| ENST00000299134.5 \| SV2 related protein homolog (rat) \| \| KIAA0430 \| ENST00000396368.3 \| KIAA0430 \| \| C1QL1 \| ENST00000253407.3 \| complement component 1, q subcomponent-like 1 \| \| BCL2L1 \| ENST00000376062.2 \| BCL2-like 1 \| \| GDI2 \| ENST00000380191.4 \| GDP dissociation inhibitor 2 \| \| PRKCB \| ENST00000303531.7 \| protein kinase C, beta \| \| PURB \| ENST00000395699.2 \| purine-rich element binding protein B \| \| SLC25A39 \| ENST00000225308.8 \| solute carrier family 25, member 39 \| \| IL6ST \| ENST00000381287.4 \| interleukin 6 signal transducer (gp130, oncostatin M receptor) \| \| EFNA4 \| ENST00000368409.3 \| ephrin-A4 \| \| MYH9 \| ENST00000216181.5 \| myosin, heavy chain 9, non-muscle \| \| TENM1 \| ENST00000371130.3 \| teneurin transmembrane protein 1 \| \| SLC4A1 \| ENST00000262418.6 \| solute carrier family 4 (anion exchanger), member 1 \| \| CNKSR2 \| ENST00000379510.3 \| connector enhancer of kinase suppressor of Ras 2 \| \| NIPA2 \| ENST00000337451.3 \| non imprinted in Prader-Willi/Angelman syndrome 2 \| \| DNAJB1 \| ENST00000254322.2 \| DnaJ (Hsp40) homolog, subfamily B, member 1 \| \| RWDD2A \| ENST00000369724.4 \| RWD domain containing 2A \| \| FAM49A \| ENST00000381323.3 \| family with sequence similarity 49, member A \| \| ENC1 \| ENST00000302351.4 \| ectodermal-neural cortex 1 (with BTB domain) \| \| SLC7A8 \| ENST00000316902.7 \| solute carrier family 7 (amino acid transporter light chain, L system), member 8 \| \| KLHL42 \| ENST00000381271.2 \| kelch-like family member 42 \| \| MTMR4 \| ENST00000579925.1 \| myotubularin related protein 4 \| \| FAM43B \| ENST00000332947.4 \| family with sequence similarity 43, member B \| \| UBE2K \| ENST00000261427.5 \| ubiquitin-conjugating enzyme E2K \| \| ANKRD12 \| ENST00000262126.4 \| ankyrin repeat domain 12 \| \| TK2 \| ENST00000299697.7 \| thymidine kinase 2, mitochondrial \| \| B3GNT9 \| ENST00000449549.3 \| UDP-GlcNAc:betaGal beta-1,3-N-acetylglucosaminyltransferase 9 \| \| AQP1 \| ENST00000311813.4 \| aquaporin 1 \| \| SOGA1 \| ENST00000279034.6 \| suppressor of glucose, autophagy associated 1 \| \| DYNC1LI2 \| ENST00000258198.2 \| dynein, cytoplasmic 1, light intermediate chain 2 \| \| GPR173 \| ENST00000332582.4 \| G protein-coupled receptor 173 \| \| FLVCR1 \| ENST00000366971.4 \| feline leukemia virus subgroup C cellular receptor 1 \| \| SCOC \| ENST00000608372.1 \| short coiled-coil protein \| \| KPNA6 \| ENST00000373625.3 \| karyopherin alpha 6 (importin alpha 7) \| \| TPD52 \| ENST00000379096.5 \| tumor protein D52 \| \| FAM193B \| ENST00000514747.1 \| family with sequence similarity 193, member B \| \| TMEM158 \| ENST00000503771.1 \| transmembrane protein 158 (gene/pseudogene) \| \| ADRA2B \| ENST00000409345.3 \| adrenoceptor alpha 2B \| \| AP1B1 \| ENST00000357586.2 \| adaptor-related protein complex 1, beta 1 subunit \| \| POLH \| ENST00000372226.1 \| polymerase (DNA directed), eta \| \| MAML3 \| ENST00000509479.2 \| mastermind-like 3 (Drosophila) \| \| CKAP4 \| ENST00000378026.4 \| cytoskeleton-associated protein 4 \| \| TTYH3 \| ENST00000258796.7 \| tweety family member 3 \| \| TRIM56 \| ENST00000306085.6 \| tripartite motif containing 56 \| \| IDH1 \| ENST00000345146.2 \| isocitrate dehydrogenase 1 (NADP+), soluble \| \| CDIP1 \| ENST00000564828.1 \| cell death-inducing p53 target 1 \| \| AFAP1 \| ENST00000360265.4 \| actin filament associated protein 1 \| \| KLHL9 \| ENST00000359039.4 \| kelch-like family member 9 \| \| SLC41A1 \| ENST00000367137.3 \| solute carrier family 41 (magnesium transporter), member 1 \| \| ERMP1 \| ENST00000381506.3 \| endoplasmic reticulum metallopeptidase 1 \| \| GDI1 \| ENST00000447750.2 \| GDP dissociation inhibitor 1 \| \| B3GNT2 \| ENST00000301998.4 \| UDP-GlcNAc:betaGal beta-1,3-N-acetylglucosaminyltransferase 2 \| \| DLGAP3 \| ENST00000373347.1 \| discs, large (Drosophila) homolog-associated protein 3 \| \| GCLC \| ENST00000229416.6 \| glutamate-cysteine ligase, catalytic subunit \| \| DIAPH2 \| ENST00000324765.8 \| diaphanous-related formin 2 \| \| CREB5 \| ENST00000357727.2 \| cAMP responsive element binding protein 5 \| \| CACUL1 \| ENST00000369151.3 \| CDK2-associated, cullin domain 1 \| \| EEF1A1 \| ENST00000316292.9 \| eukaryotic translation elongation factor 1 alpha 1 \| \| TFE3 \| ENST00000315869.7 \| transcription factor binding to IGHM enhancer 3 \| \| NCAN \| ENST00000252575.6 \| neurocan \| \| WNT4 \| ENST00000290167.6 \| wingless-type MMTV integration site family, member 4 \| \| LZTS2 \| ENST00000370223.3 \| leucine zipper, putative tumor suppressor 2 \| \| SLC8A2 \| ENST00000236877.6 \| solute carrier family 8 (sodium/calcium exchanger), member 2 \| \| GRM7 \| ENST00000486284.1 \| glutamate receptor, metabotropic 7 \| \| LETMD1 \| ENST00000380123.2 \| LETM1 domain containing 1 \| \| CCDC144A \| ENST00000443444.2 \| coiled-coil domain containing 144A \| \| ZNF436 \| ENST00000314011.4 \| zinc finger protein 436 \| \| SESN1 \| ENST00000436639.2 \| sestrin 1 \| \| FRS2 \| ENST00000550389.1 \| fibroblast growth factor receptor substrate 2 \| \| SLC7A2 \| ENST00000494857.1 \| solute carrier family 7 (cationic amino acid transporter, y+ system), member 2 \| \| MAP3K3 \| ENST00000361357.3 \| mitogen-activated protein kinase kinase kinase 3 \| \| GRIA2 \| ENST00000296526.7 \| glutamate receptor, ionotropic, AMPA 2 \| \| IQGAP2 \| ENST00000274364.6 \| IQ motif containing GTPase activating protein 2 \| \| TMEM110 \| ENST00000355083.5 \| transmembrane protein 110 \| \| PRDM16 \| ENST00000270722.5 \| PR domain containing 16 \| \| AP4E1 \| ENST00000261842.5 \| adaptor-related protein complex 4, epsilon 1 subunit \| \| UNC13A \| ENST00000519716.2 \| unc-13 homolog A (C. elegans) \| \| KLHDC10 \| ENST00000335420.5 \| kelch domain containing 10 \| \| FSCN1 \| ENST00000382361.3 \| fascin homolog 1, actin-bundling protein (Strongylocentrotus purpuratus) \| \| HS3ST5 \| ENST00000312719.5 \| heparan sulfate (glucosamine) 3-O-sulfotransferase 5 \| \| AP2M1 \| ENST00000382456.3 \| adaptor-related protein complex 2, mu 1 subunit \| \| MMP14 \| ENST00000311852.6 \| matrix metallopeptidase 14 (membrane-inserted) \| \| ANKRD52 \| ENST00000267116.7 \| ankyrin repeat domain 52 \| \| FAM160B1 \| ENST00000369248.4 \| family with sequence similarity 160, member B1 \| \| FAF2 \| ENST00000261942.6 \| Fas associated factor family member 2 \| \| ASH1L \| ENST00000368346.3 \| ash1 (absent, small, or homeotic)-like (Drosophila) \| \| FURIN \| ENST00000268171.3 \| furin (paired basic amino acid cleaving enzyme) \| \| FOXG1 \| ENST00000382535.3 \| forkhead box G1 \| \| MMP15 \| ENST00000219271.3 \| matrix metallopeptidase 15 (membrane-inserted) \| \| ABCD2 \| ENST00000308666.3 \| ATP-binding cassette, sub-family D (ALD), member 2 \| \| TGFBR1 \| ENST00000374994.4 \| transforming growth factor, beta receptor 1 \| \| SLC30A5 \| ENST00000396591.3 \| solute carrier family 30 (zinc transporter), member 5 \| \| ZBTB14 \| ENST00000357006.4 \| zinc finger and BTB domain containing 14 \| \| POU4F1 \| ENST00000377208.5 \| POU class 4 homeobox 1 \| \| SMIM14 \| ENST00000295958.5 \| small integral membrane protein 14 \| \| CDK5R1 \| ENST00000313401.3 \| cyclin-dependent kinase 5, regulatory subunit 1 (p35) \| \| KLHL11 \| ENST00000319121.3 \| kelch-like family member 11 \| \| COL1A1 \| ENST00000225964.5 \| collagen, type I, alpha 1 \| \| MLXIP \| ENST00000319080.7 \| MLX interacting protein \| \| FAM19A5 \| ENST00000358295.5 \| family with sequence similarity 19 (chemokine (C-C motif)-like), member A5 \| \| PDIK1L \| ENST00000374271.4 \| PDLIM1 interacting kinase 1 like \| \| EIF3J \| ENST00000261868.5 \| eukaryotic translation initiation factor 3, subunit J \| \| CPLX2 \| ENST00000359546.4 \| complexin 2 \| \| DLG2 \| ENST00000398309.2 \| discs, large homolog 2 (Drosophila) \| \| MRFAP1 \| ENST00000382581.4 \| Morf4 family associated protein 1 \| \| PTBP3 \| ENST00000374257.1 \| polypyrimidine tract binding protein 3 \| \| MAN1A2 \| ENST00000356554.3 \| mannosidase, alpha, class 1A, member 2 \| \| KLHL2 \| ENST00000538127.1 \| kelch-like family member 2 \| \| HOXD1 \| ENST00000331462.4 \| homeobox D1 \| \| NCKAP5L \| ENST00000335999.6 \| NCK-associated protein 5-like \| \| IFIT2 \| ENST00000371826.3 \| interferon-induced protein with tetratricopeptide repeats 2 \| \| KLF7 \| ENST00000309446.6 \| Kruppel-like factor 7 (ubiquitous) \| \| PIK3C2A \| ENST00000265970.7 \| phosphatidylinositol-4-phosphate 3-kinase, catalytic subunit type 2 alpha \| \| EGFR \| ENST00000275493.2 \| epidermal growth factor receptor \| \| ORAI2 \| ENST00000356387.2 \| ORAI calcium release-activated calcium modulator 2 \| \| UBFD1 \| ENST00000395878.3 \| ubiquitin family domain containing 1 \| \| CCDC30 \| ENST00000342022.4 \| coiled-coil domain containing 30 \| \| ADAMTS5 \| ENST00000284987.5 \| ADAM metallopeptidase with thrombospondin type 1 motif, 5 \| \| PWWP2B \| ENST00000305233.5 \| PWWP domain containing 2B \| \| HSPA13 \| ENST00000285667.3 \| heat shock protein 70kDa family, member 13 \| \| EYA1 \| ENST00000388742.4 \| eyes absent homolog 1 (Drosophila) \| \| CSNK1G3 \| ENST00000360683.2 \| casein kinase 1, gamma 3 \| \| NTNG1 \| ENST00000370067.1 \| netrin G1 \| \| CDYL2 \| ENST00000570137.2 \| chromodomain protein, Y-like 2 \| \| HLF \| ENST00000226067.5 \| hepatic leukemia factor \| \| SLC25A51 \| ENST00000380590.3 \| solute carrier family 25, member 51 \| \| ZNF395 \| ENST00000344423.5 \| zinc finger protein 395 \| \| NCAM2 \| ENST00000400546.1 \| neural cell adhesion molecule 2 \| \| CDCA8 \| ENST00000373055.1 \| cell division cycle associated 8 \| \| PPP6R3 \| ENST00000393800.2 \| protein phosphatase 6, regulatory subunit 3 \| \| NELFA \| ENST00000382882.3 \| negative elongation factor complex member A \| \| ZHX1 \| ENST00000395571.3 \| zinc fingers and homeoboxes 1 \| \| ITPRIPL2 \| ENST00000381440.3 \| inositol 1,4,5-trisphosphate receptor interacting protein-like 2 \| \| TRHDE \| ENST00000261180.4 \| thyrotropin-releasing hormone degrading enzyme \| \| PCDH15 \| ENST00000395438.1 \| protocadherin-related 15 \| \| USH1G \| ENST00000319642.1 \| Usher syndrome 1G (autosomal recessive) \| \| FAM13A \| ENST00000395002.2 \| family with sequence similarity 13, member A \| \| ARX \| ENST00000379044.4 \| aristaless related homeobox \| \| NELL2 \| ENST00000395487.2 \| NEL-like 2 (chicken) \| \| NEBL \| ENST00000377122.4 \| nebulette \| \| KCNJ12 \| ENST00000583088.1 \| potassium inwardly-rectifying channel, subfamily J, member 12 \| \| ADCY6 \| ENST00000357869.3 \| adenylate cyclase 6 \| \| WTAP \| ENST00000358372.4 \| Wilms tumor 1 associated protein \| \| OTUD7B \| ENST00000369135.4 \| OTU domain containing 7B \| \| BAZ2A \| ENST00000379441.3 \| bromodomain adjacent to zinc finger domain, 2A \| \| ELMO1 \| ENST00000341056.3 \| engulfment and cell motility 1 \| \| SLC26A2 \| ENST00000286298.4 \| solute carrier family 26 (anion exchanger), member 2 \| \| EPS15 \| ENST00000371730.2 \| epidermal growth factor receptor pathway substrate 15 \| \| ESYT1 \| ENST00000394048.5 \| extended synaptotagmin-like protein 1 \| \| PPP2R4 \| ENST00000393370.2 \| protein phosphatase 2A activator, regulatory subunit 4 \| \| WDR59 \| ENST00000262144.6 \| WD repeat domain 59 \| \| PARD6G \| ENST00000353265.3 \| par-6 family cell polarity regulator gamma \| \| RFX3 \| ENST00000382004.3 \| regulatory factor X, 3 (influences HLA class II expression) \| \| USP6 \| ENST00000250066.6 \| ubiquitin specific peptidase 6 (Tre-2 oncogene) \| \| LIN7A \| ENST00000552864.1 \| lin-7 homolog A (C. elegans) \| \| DOT1L \| ENST00000398665.3 \| DOT1-like histone H3K79 methyltransferase \| \| PCDH17 \| ENST00000377918.3 \| protocadherin 17 \| \| PPP2R5E \| ENST00000337537.3 \| protein phosphatase 2, regulatory subunit B', epsilon isoform \| \| NR6A1 \| ENST00000487099.2 \| nuclear receptor subfamily 6, group A, member 1 \| \| SLC27A4 \| ENST00000300456.4 \| solute carrier family 27 (fatty acid transporter), member 4 \| \| PDE10A \| ENST00000366882.1 \| phosphodiesterase 10A \| \| SFXN5 \| ENST00000272433.2 \| sideroflexin 5 \| \| RHOA \| ENST00000454011.2 \| ras homolog family member A \| \| DDX19B \| ENST00000288071.6 \| DEAD (Asp-Glu-Ala-Asp) box polypeptide 19B \| \| MAFG \| ENST00000357736.4 \| v-maf avian musculoaponeurotic fibrosarcoma oncogene homolog G \| \| MRGBP \| ENST00000370487.3 \| MRG/MORF4L binding protein \| \| PSD3 \| ENST00000327040.8 \| pleckstrin and Sec7 domain containing 3 \| \| XPO1 \| ENST00000401558.2 \| exportin 1 (CRM1 homolog, yeast) \| \| KCNK7 \| ENST00000394216.2 \| potassium channel, subfamily K, member 7 \| \| SPOPL \| ENST00000280098.4 \| speckle-type POZ protein-like \| \| SFT2D3 \| ENST00000310981.4 \| SFT2 domain containing 3 \| \| C17orf96 \| ENST00000325814.5 \| chromosome 17 open reading frame 96 \| \| FOXP2 \| ENST00000408937.3 \| forkhead box P2 \| \| AK2 \| ENST00000467905.1 \| adenylate kinase 2 \| \| DBNL \| ENST00000494774.1 \| drebrin-like \| \| LIN7C \| ENST00000278193.2 \| lin-7 homolog C (C. elegans) \| \| PTMA \| ENST00000409115.3 \| prothymosin, alpha \| \| DSTYK \| ENST00000367160.4 \| dual serine/threonine and tyrosine protein kinase \| \| MTX3 \| ENST00000509852.1 \| metaxin 3 \| \| PCSK6 \| ENST00000348070.1 \| proprotein convertase subtilisin/kexin type 6 \| \| UNK \| ENST00000293218.3 \| unkempt homolog (Drosophila) \| \| CNTNAP1 \| ENST00000264638.4 \| contactin associated protein 1 \| \| NRG3 \| ENST00000372142.2 \| neuregulin 3 \| \| ZBTB4 \| ENST00000380599.4 \| zinc finger and BTB domain containing 4 \| \| USP38 \| ENST00000307017.4 \| ubiquitin specific peptidase 38 \| \| SMPD4 \| ENST00000351288.6 \| sphingomyelin phosphodiesterase 4, neutral membrane (neutral sphingomyelinase-3) \| \| MID2 \| ENST00000262843.6 \| midline 2 \| \| SCN1A \| ENST00000423058.2 \| sodium channel, voltage-gated, type I, alpha subunit \| \| MMGT1 \| ENST00000305963.2 \| membrane magnesium transporter 1 \| \| TRIM55 \| ENST00000315962.4 \| tripartite motif containing 55 \| \| CD47 \| ENST00000361309.5 \| CD47 molecule \| \| EYA4 \| ENST00000367895.5 \| eyes absent homolog 4 (Drosophila) \| \| GALNT16 \| ENST00000337827.4 \| UDP-N-acetyl-alpha-D-galactosamine:polypeptide N-acetylgalactosaminyltransferase 16 \| \| LRRC2 \| ENST00000395905.3 \| leucine rich repeat containing 2 \| \| PDK3 \| ENST00000441463.2 \| pyruvate dehydrogenase kinase, isozyme 3 \| \| KIAA0232 \| ENST00000425103.1 \| KIAA0232 \| \| AMD1 \| ENST00000368885.3 \| adenosylmethionine decarboxylase 1 \| \| PCSK5 \| ENST00000376752.4 \| proprotein convertase subtilisin/kexin type 5 \| \| LTBP1 \| ENST00000404525.1 \| latent transforming growth factor beta binding protein 1 \| \| AIF1L \| ENST00000372300.1 \| allograft inflammatory factor 1-like \| \| DDX3Y \| ENST00000336079.3 \| DEAD (Asp-Glu-Ala-Asp) box helicase 3, Y-linked \| \| GABBR2 \| ENST00000259455.2 \| gamma-aminobutyric acid (GABA) B receptor, 2 \| \| KIF21A \| ENST00000361961.3 \| kinesin family member 21A \| \| SIX5 \| ENST00000560168.1 \| SIX homeobox 5 \| \| SIRT1 \| ENST00000212015.6 \| sirtuin 1 \| \| PEAK1 \| ENST00000312493.4 \| pseudopodium-enriched atypical kinase 1 \| \| SMC2 \| ENST00000374793.3 \| structural maintenance of chromosomes 2 \| \| PHTF2 \| ENST00000416283.2 \| putative homeodomain transcription factor 2 \| \| HIVEP2 \| ENST00000367604.1 \| human immunodeficiency virus type I enhancer binding protein 2 \| \| SYNPO2L \| ENST00000372873.4 \| synaptopodin 2-like \| \| ESRRG \| ENST00000361525.3 \| estrogen-related receptor gamma \| \| ZNF217 \| ENST00000371471.2 \| zinc finger protein 217 \| \| HLTF \| ENST00000465259.1 \| helicase-like transcription factor \| \| ZBTB38 \| ENST00000514251.1 \| zinc finger and BTB domain containing 38 \| \| TAOK1 \| ENST00000261716.3 \| TAO kinase 1 \| \| ARHGAP39 \| ENST00000377307.2 \| Rho GTPase activating protein 39 \| \| EPHB4 \| ENST00000360620.3 \| EPH receptor B4 \| \| HOXB5 \| ENST00000239151.5 \| homeobox B5 \| \| LPGAT1 \| ENST00000366997.4 \| lysophosphatidylglycerol acyltransferase 1 \| \| JAZF1 \| ENST00000283928.5 \| JAZF zinc finger 1 \| \| ZNF362 \| ENST00000539719.1 \| zinc finger protein 362 \| \| DNAJC3 \| ENST00000602402.1 \| DnaJ (Hsp40) homolog, subfamily C, member 3 \| \| ZBTB10 \| ENST00000430430.1 \| zinc finger and BTB domain containing 10 \| \| ZNF664 \| ENST00000538932.2 \| zinc finger protein 664 \| \| GID8 \| ENST00000266069.3 \| GID complex subunit 8 \| \| MYO9B \| ENST00000595618.1 \| myosin IXB \| \| INSR \| ENST00000341500.5 \| insulin receptor \| \| C2orf69 \| ENST00000319974.5 \| chromosome 2 open reading frame 69 \| \| KIAA1522 \| ENST00000401073.2 \| KIAA1522 \| \| CHRM3 \| ENST00000255380.4 \| cholinergic receptor, muscarinic 3 \| \| YIPF4 \| ENST00000238831.4 \| Yip1 domain family, member 4 \| \| NFYA \| ENST00000341376.6 \| nuclear transcription factor Y, alpha \| \| E2F7 \| ENST00000322886.7 \| E2F transcription factor 7 \| \| SLC33A1 \| ENST00000392845.3 \| solute carrier family 33 (acetyl-CoA transporter), member 1 \| \| SMARCA2 \| ENST00000349721.2 \| SWI/SNF related, matrix associated, actin dependent regulator of chromatin, subfamily a, member 2 \| \| CSRNP1 \| ENST00000273153.5 \| cysteine-serine-rich nuclear protein 1 \| \| FAM199X \| ENST00000493442.1 \| family with sequence similarity 199, X-linked \| \| NUS1 \| ENST00000368494.3 \| nuclear undecaprenyl pyrophosphate synthase 1 homolog (S. cerevisiae) \| \| SPRY4 \| ENST00000344120.4 \| sprouty homolog 4 (Drosophila) \| \| DUSP7 \| ENST00000495880.1 \| dual specificity phosphatase 7 \| \| KIAA0141 \| ENST00000194118.4 \| KIAA0141 \| \| GNB4 \| ENST00000232564.3 \| guanine nucleotide binding protein (G protein), beta polypeptide 4 \| \| ACTR1A \| ENST00000487599.1 \| ARP1 actin-related protein 1 homolog A, centractin alpha (yeast) \| \| UBE2Z \| ENST00000360943.5 \| ubiquitin-conjugating enzyme E2Z \| \| THUMPD3 \| ENST00000345094.3 \| THUMP domain containing 3 \| \| RFX6 \| ENST00000332958.2 \| regulatory factor X, 6 \| \| SPTSSA \| ENST00000298130.4 \| serine palmitoyltransferase, small subunit A \| \| TBL1X \| ENST00000407597.2 \| transducin (beta)-like 1X-linked \| \| ALDH5A1 \| ENST00000357578.3 \| aldehyde dehydrogenase 5 family, member A1 \| \| TET3 \| ENST00000409262.3 \| tet methylcytosine dioxygenase 3 \| \| CNNM2 \| ENST00000369878.4 \| cyclin M2 \| \| VAMP3 \| ENST00000054666.6 \| vesicle-associated membrane protein 3 \| \| SETD6 \| ENST00000394266.4 \| SET domain containing 6 \| \| ZNRF3 \| ENST00000544604.2 \| zinc and ring finger 3 \| \| FBN1 \| ENST00000316623.5 \| fibrillin 1 \| \| SGSM1 \| ENST00000400358.4 \| small G protein signaling modulator 1 \| \| SORBS3 \| ENST00000240123.7 \| sorbin and SH3 domain containing 3 \| \| LZTS3 \| ENST00000329152.3 \| Homo sapiens leucine zipper, putative tumor suppressor family member 3 (LZTS3), transcript variant 2, mRNA. \| \| MIER3 \| ENST00000381226.3 \| mesoderm induction early response 1, family member 3 \| \| CNOT1 \| ENST00000317147.5 \| CCR4-NOT transcription complex, subunit 1 \| \| ANAPC1 \| ENST00000341068.3 \| anaphase promoting complex subunit 1 \| \| UNC80 \| ENST00000439458.1 \| unc-80 homolog (C. elegans) \| \| RAB5C \| ENST00000346213.4 \| RAB5C, member RAS oncogene family \| \| ABL2 \| ENST00000502732.1 \| c-abl oncogene 2, non-receptor tyrosine kinase \| \| ZNF385A \| ENST00000551109.1 \| zinc finger protein 385A \| \| SYT9 \| ENST00000318881.6 \| synaptotagmin IX \| \| IMPG1 \| ENST00000369963.3 \| interphotoreceptor matrix proteoglycan 1 \| \| TOB2 \| ENST00000327492.3 \| transducer of ERBB2, 2 \| \| SYAP1 \| ENST00000380155.3 \| synapse associated protein 1 \| \| FAM73A \| ENST00000370791.3 \| family with sequence similarity 73, member A \| \| CUL4B \| ENST00000371322.5 \| cullin 4B \| \| ABCC1 \| ENST00000399408.2 \| ATP-binding cassette, sub-family C (CFTR/MRP), member 1 \| \| ANK2 \| ENST00000357077.4 \| ankyrin 2, neuronal \| \| RAB11FIP4 \| ENST00000325874.8 \| RAB11 family interacting protein 4 (class II) \| \| WDTC1 \| ENST00000319394.3 \| WD and tetratricopeptide repeats 1 \| \| FBN2 \| ENST00000262464.4 \| fibrillin 2 \| \| PDE7A \| ENST00000401827.3 \| phosphodiesterase 7A \| \| DCUN1D4 \| ENST00000334635.5 \| DCN1, defective in cullin neddylation 1, domain containing 4 \| \| TMEM33 \| ENST00000504986.1 \| transmembrane protein 33 \| \| KMT2C \| ENST00000262189.6 \| lysine (K)-specific methyltransferase 2C \| \| ZBTB16 \| ENST00000335953.4 \| zinc finger and BTB domain containing 16 \| \| NFIB \| ENST00000397575.3 \| nuclear factor I/B \| \| LIN28B \| ENST00000345080.4 \| lin-28 homolog B (C. elegans) \| \| SPTBN1 \| ENST00000356805.4 \| spectrin, beta, non-erythrocytic 1 \| \| KIAA1549 \| ENST00000440172.1 \| KIAA1549 \| \| TRAF3IP2 \| ENST00000368761.5 \| TRAF3 interacting protein 2 \| \| MPPED1 \| ENST00000417669.2 \| metallophosphoesterase domain containing 1 \| \| ABCG4 \| ENST00000307417.3 \| ATP-binding cassette, sub-family G (WHITE), member 4 \| \| VPS4B \| ENST00000238497.5 \| vacuolar protein sorting 4 homolog B (S. cerevisiae) \| \| ENTPD7 \| ENST00000370489.4 \| ectonucleoside triphosphate diphosphohydrolase 7 \| \| VTI1B \| ENST00000554659.1 \| vesicle transport through interaction with t-SNAREs 1B \| \| NMT1 \| ENST00000592782.1 \| N-myristoyltransferase 1 \| \| HMGXB3 \| ENST00000503427.1 \| HMG box domain containing 3 \| \| MRC2 \| ENST00000303375.5 \| mannose receptor, C type 2 \| \| MED8 \| ENST00000372457.4 \| mediator complex subunit 8 \| \| PARVA \| ENST00000334956.8 \| parvin, alpha \| \| NFIA \| ENST00000403491.3 \| nuclear factor I/A \| \| STARD13 \| ENST00000336934.5 \| StAR-related lipid transfer (START) domain containing 13 \| \| ARHGAP24 \| ENST00000395184.1 \| Rho GTPase activating protein 24 \| \| SRP19 \| ENST00000282999.3 \| signal recognition particle 19kDa \| \| FAM102B \| ENST00000370035.3 \| family with sequence similarity 102, member B \| \| C11orf58 \| ENST00000228136.4 \| chromosome 11 open reading frame 58 \| \| CYLD \| ENST00000540145.1 \| cylindromatosis (turban tumor syndrome) \| \| TNRC6B \| ENST00000335727.9 \| trinucleotide repeat containing 6B \| \| GDF11 \| ENST00000257868.5 \| growth differentiation factor 11 \| \| SLC30A3 \| ENST00000233535.4 \| solute carrier family 30 (zinc transporter), member 3 \| \| RP1-170O19.20 \| ENST00000470747.4 \| Uncharacterized protein \| \| CCNT1 \| ENST00000261900.3 \| cyclin T1 \| \| TYRO3 \| ENST00000263798.3 \| TYRO3 protein tyrosine kinase \| \| KIAA1239 \| ENST00000309447.5 \| KIAA1239 \| \| ARHGDIA \| ENST00000269321.7 \| Rho GDP dissociation inhibitor (GDI) alpha \| \| LUZP1 \| ENST00000418342.1 \| leucine zipper protein 1 \| \| TMEM87B \| ENST00000283206.4 \| transmembrane protein 87B \| \| NKD1 \| ENST00000268459.3 \| naked cuticle homolog 1 (Drosophila) \| \| RAB27B \| ENST00000262094.5 \| RAB27B, member RAS oncogene family \| \| HOXA9 \| ENST00000396345.1 \| homeobox A9 \| \| BNC2 \| ENST00000380672.4 \| basonuclin 2 \| \| ZNF354B \| ENST00000322434.3 \| zinc finger protein 354B \| \| TNIK \| ENST00000436636.2 \| TRAF2 and NCK interacting kinase \| \| PPP1R9B \| ENST00000316878.6 \| protein phosphatase 1, regulatory subunit 9B \| \| ADCY5 \| ENST00000462833.1 \| adenylate cyclase 5 \| \| PTPRT \| ENST00000373187.1 \| protein tyrosine phosphatase, receptor type, T \| \| SP1 \| ENST00000426431.2 \| Sp1 transcription factor \| \| AAK1 \| ENST00000409085.4 \| AP2 associated kinase 1 \| \| GIGYF1 \| ENST00000275732.5 \| GRB10 interacting GYF protein 1 \| \| BHLHE41 \| ENST00000242728.4 \| basic helix-loop-helix family, member e41 \| \| CHD2 \| ENST00000394196.4 \| chromodomain helicase DNA binding protein 2 \| \| CGGBP1 \| ENST00000309534.6 \| CGG triplet repeat binding protein 1 \| \| SF3B1 \| ENST00000335508.6 \| splicing factor 3b, subunit 1, 155kDa \| \| NFAT5 \| ENST00000354436.2 \| nuclear factor of activated T-cells 5, tonicity-responsive \| \| ID4 \| ENST00000378700.3 \| inhibitor of DNA binding 4, dominant negative helix-loop-helix protein \| \| OTUD4 \| ENST00000454497.2 \| OTU domain containing 4 \| \| UCK2 \| ENST00000367879.4 \| uridine-cytidine kinase 2 \| \| CUX2 \| ENST00000261726.6 \| cut-like homeobox 2 \| \| CAPN5 \| ENST00000278559.3 \| calpain 5 \| \| TGOLN2 \| ENST00000377386.3 \| trans-golgi network protein 2 \| \| CNNM4 \| ENST00000540067.1 \| cyclin M4 \| \| IGF1R \| ENST00000268035.6 \| insulin-like growth factor 1 receptor \| \| LEPROTL1 \| ENST00000321250.8 \| leptin receptor overlapping transcript-like 1 \| \| USP32 \| ENST00000300896.4 \| ubiquitin specific peptidase 32 \| \| FAM208A \| ENST00000493960.2 \| family with sequence similarity 208, member A \| \| ARRB1 \| ENST00000420843.2 \| arrestin, beta 1 \| \| FAM46C \| ENST00000369448.3 \| family with sequence similarity 46, member C \| \| ATRX \| ENST00000373344.5 \| alpha thalassemia/mental retardation syndrome X-linked \| \| PROX1 \| ENST00000366958.4 \| prospero homeobox 1 \| \| MKL2 \| ENST00000318282.5 \| MKL/myocardin-like 2 \| \| NIPA1 \| ENST00000337435.4 \| non imprinted in Prader-Willi/Angelman syndrome 1 \| \| KIAA1024 \| ENST00000305428.3 \| KIAA1024 \| \| SH3PXD2B \| ENST00000311601.5 \| SH3 and PX domains 2B \| \| ADCYAP1 \| ENST00000579794.1 \| adenylate cyclase activating polypeptide 1 (pituitary) \| \| DMXL1 \| ENST00000311085.8 \| Dmx-like 1 \| \| PITPNM3 \| ENST00000421306.3 \| PITPNM family member 3 \| \| SRSF10 \| ENST00000343255.5 \| serine/arginine-rich splicing factor 10 \| \| TRAF3 \| ENST00000560371.1 \| TNF receptor-associated factor 3 \| \| ICK \| ENST00000350082.5 \| intestinal cell (MAK-like) kinase \| \| ARNTL2 \| ENST00000546179.1 \| aryl hydrocarbon receptor nuclear translocator-like 2 \| \| NUP153 \| ENST00000262077.2 \| nucleoporin 153kDa \| \| HECW1 \| ENST00000395891.2 \| HECT, C2 and WW domain containing E3 ubiquitin protein ligase 1 \| \| SFMBT2 \| ENST00000361972.4 \| Scm-like with four mbt domains 2 \| \| MFSD6 \| ENST00000392328.1 \| major facilitator superfamily domain containing 6 \| \| ZC3H14 \| ENST00000251038.5 \| zinc finger CCCH-type containing 14 \| \| PAWR \| ENST00000328827.4 \| PRKC, apoptosis, WT1, regulator \| \| PANK3 \| ENST00000239231.6 \| pantothenate kinase 3 \| \| PCDH1 \| ENST00000503492.1 \| protocadherin 1 \| \| SPTLC2 \| ENST00000216484.2 \| serine palmitoyltransferase, long chain base subunit 2 \| \| FOXK2 \| ENST00000335255.5 \| forkhead box K2 \| \| NLGN2 \| ENST00000302926.2 \| neuroligin 2 \| \| DDX3X \| ENST00000399959.2 \| DEAD (Asp-Glu-Ala-Asp) box helicase 3, X-linked \| \| RRP15 \| ENST00000366932.3 \| ribosomal RNA processing 15 homolog (S. cerevisiae) \| \| RLN2 \| ENST00000308420.3 \| relaxin 2 \| \| ATP2A2 \| ENST00000395494.2 \| ATPase, Ca++ transporting, cardiac muscle, slow twitch 2 \| \| SRPK1 \| ENST00000373825.2 \| SRSF protein kinase 1 \| \| ISCA2 \| ENST00000554924.1 \| iron-sulfur cluster assembly 2 \| \| SDC2 \| ENST00000302190.4 \| syndecan 2 \| \| RTN4RL1 \| ENST00000331238.6 \| reticulon 4 receptor-like 1 \| \| LPHN1 \| ENST00000340736.6 \| latrophilin 1 \| \| HIC2 \| ENST00000407464.2 \| hypermethylated in cancer 2 \| \| PPM1H \| ENST00000228705.6 \| protein phosphatase, Mg2+/Mn2+ dependent, 1H \| \| MCL1 \| ENST00000369026.2 \| myeloid cell leukemia sequence 1 (BCL2-related) \| \| ZHX3 \| ENST00000309060.3 \| zinc fingers and homeoboxes 3 \| \| ADARB2 \| ENST00000381312.1 \| adenosine deaminase, RNA-specific, B2 (non-functional) \| \| MECP2 \| ENST00000303391.6 \| methyl CpG binding protein 2 (Rett syndrome) \| \| FOXP1 \| ENST00000318789.4 \| forkhead box P1 \| \| FBXL19 \| ENST00000338343.4 \| F-box and leucine-rich repeat protein 19 \| \| CALM1 \| ENST00000356978.4 \| calmodulin 1 (phosphorylase kinase, delta) \| \| DESI1 \| ENST00000263256.6 \| desumoylating isopeptidase 1 \| \| ASB1 \| ENST00000264607.4 \| ankyrin repeat and SOCS box containing 1 \| \| ACAT2 \| ENST00000541436.1 \| acetyl-CoA acetyltransferase 2 \| \| ZBTB7B \| ENST00000368426.3 \| zinc finger and BTB domain containing 7B \| \| FARP1 \| ENST00000595437.1 \| FERM, RhoGEF (ARHGEF) and pleckstrin domain protein 1 (chondrocyte-derived) \| \| ESYT2 \| ENST00000251527.5 \| extended synaptotagmin-like protein 2 \| \| HSPA4L \| ENST00000296464.4 \| heat shock 70kDa protein 4-like \| \| SRGAP3 \| ENST00000383836.3 \| SLIT-ROBO Rho GTPase activating protein 3 \| \| WIPF2 \| ENST00000323571.4 \| WAS/WASL interacting protein family, member 2 \| \| LGALS8 \| ENST00000526589.1 \| lectin, galactoside-binding, soluble, 8 \| \| FST \| ENST00000256759.3 \| follistatin \| \| HHIP \| ENST00000296575.3 \| hedgehog interacting protein \| \| SLC8A1 \| ENST00000406785.2 \| solute carrier family 8 (sodium/calcium exchanger), member 1 \| \| BNIP3L \| ENST00000380629.2 \| BCL2/adenovirus E1B 19kDa interacting protein 3-like \| \| PKNOX1 \| ENST00000291547.5 \| PBX/knotted 1 homeobox 1 \| \| KCTD16 \| ENST00000507359.3 \| potassium channel tetramerization domain containing 16 \| \| SESN3 \| ENST00000536441.1 \| sestrin 3 \| \| UNKL \| ENST00000389221.4 \| unkempt homolog (Drosophila)-like \| \| FOXN3 \| ENST00000345097.4 \| forkhead box N3 \| \| BAMBI \| ENST00000375533.3 \| BMP and activin membrane-bound inhibitor \| \| PIP4K2B \| ENST00000269554.3 \| phosphatidylinositol-5-phosphate 4-kinase, type II, beta \| \| CACNA1B \| ENST00000277551.2 \| calcium channel, voltage-dependent, N type, alpha 1B subunit \| \| RPAP2 \| ENST00000610020.1 \| RNA polymerase II associated protein 2 \| \| CHD3 \| ENST00000380358.4 \| chromodomain helicase DNA binding protein 3 \| \| CCNDBP1 \| ENST00000300213.4 \| cyclin D-type binding-protein 1 \| \| CSTF2 \| ENST00000415585.2 \| cleavage stimulation factor, 3' pre-RNA, subunit 2, 64kDa \| \| WIPI2 \| ENST00000288828.4 \| WD repeat domain, phosphoinositide interacting 2 \| \| TROVE2 \| ENST00000432079.1 \| TROVE domain family, member 2 \| \| MRPL44 \| ENST00000258383.3 \| mitochondrial ribosomal protein L44 \| \| SPTY2D1 \| ENST00000336349.5 \| SPT2, Suppressor of Ty, domain containing 1 (S. cerevisiae) \| \| CEP128 \| ENST00000281129.3 \| centrosomal protein 128kDa \| \| PRMT6 \| ENST00000370078.1 \| protein arginine methyltransferase 6 \| \| ZNF322 \| ENST00000415922.2 \| zinc finger protein 322 \| \| ORC4 \| ENST00000392857.5 \| origin recognition complex, subunit 4 \| \| GABRB1 \| ENST00000295454.3 \| gamma-aminobutyric acid (GABA) A receptor, beta 1 \| \| AQP1 \| ENST00000509504.1 \| Uncharacterized protein \| \| ZBTB20 \| ENST00000462705.1 \| zinc finger and BTB domain containing 20 \| \| ENPP6 \| ENST00000296741.2 \| ectonucleotide pyrophosphatase/phosphodiesterase 6 \| \| TG \| ENST00000519543.1 \| thyroglobulin \| \| MAP7 \| ENST00000354570.3 \| microtubule-associated protein 7 \| |
| --- | --- | --- | --- | --- | --- | --- | --- | --- | --- | --- | --- | --- | --- | --- | --- | --- | --- | --- | --- | --- | --- | --- | --- | --- | --- | --- | --- | --- | --- | --- | --- | --- | --- | --- | --- | --- | --- | --- | --- | --- | --- | --- | --- | --- | --- | --- | --- | --- | --- | --- | --- | --- | --- | --- | --- | --- | --- | --- | --- | --- | --- | --- | --- | --- | --- | --- | --- | --- | --- | --- | --- | --- | --- | --- | --- | --- | --- | --- | --- | --- | --- | --- | --- | --- | --- | --- | --- | --- | --- | --- | --- | --- | --- | --- | --- | --- | --- | --- | --- | --- | --- | --- | --- | --- | --- | --- | --- | --- | --- | --- | --- | --- | --- | --- | --- | --- | --- | --- | --- | --- | --- | --- | --- | --- | --- | --- | --- | --- | --- | --- | --- | --- | --- | --- | --- | --- | --- | --- | --- | --- | --- | --- | --- | --- | --- | --- | --- | --- | --- | --- | --- | --- | --- | --- | --- | --- | --- | --- | --- | --- | --- | --- | --- | --- | --- | --- | --- | --- | --- | --- | --- | --- | --- | --- | --- | --- | --- | --- | --- | --- | --- | --- | --- | --- | --- | --- | --- | --- | --- | --- | --- | --- | --- | --- | --- | --- | --- | --- | --- | --- | --- | --- | --- | --- | --- | --- | --- | --- | --- | --- | --- | --- | --- | --- | --- | --- | --- | --- | --- | --- | --- | --- | --- | --- | --- | --- | --- | --- | --- | --- | --- | --- | --- | --- | --- | --- | --- | --- | --- | --- | --- | --- | --- | --- | --- | --- | --- | --- | --- | --- | --- | --- | --- | --- | --- | --- | --- | --- | --- | --- | --- | --- | --- | --- | --- | --- | --- | --- | --- | --- | --- | --- | --- | --- | --- | --- | --- | --- | --- | --- | --- | --- | --- | --- | --- | --- | --- | --- | --- | --- | --- | --- | --- | --- | --- | --- | --- | --- | --- | --- | --- | --- | --- | --- | --- | --- | --- | --- | --- | --- | --- | --- | --- | --- | --- | --- | --- | --- | --- | --- | --- | --- | --- | --- | --- | --- | --- | --- | --- | --- | --- | --- | --- | --- | --- | --- | --- | --- | --- | --- | --- | --- | --- | --- | --- | --- | --- | --- | --- | --- | --- | --- | --- | --- | --- | --- | --- | --- | --- | --- | --- | --- | --- | --- | --- | --- | --- | --- | --- | --- | --- | --- | --- | --- | --- | --- | --- | --- | --- | --- | --- | --- | --- | --- | --- | --- | --- | --- | --- | --- | --- | --- | --- | --- | --- | --- | --- | --- | --- | --- | --- | --- | --- | --- | --- | --- | --- | --- | --- | --- | --- | --- | --- | --- | --- | --- | --- | --- | --- | --- | --- | --- | --- | --- | --- | --- | --- | --- | --- | --- | --- | --- | --- | --- | --- | --- | --- | --- | --- | --- | --- | --- | --- | --- | --- | --- | --- | --- | --- | --- | --- | --- | --- | --- | --- | --- | --- | --- | --- | --- | --- | --- | --- | --- | --- | --- | --- | --- | --- | --- | --- | --- | --- | --- | --- | --- | --- | --- | --- | --- | --- | --- | --- | --- | --- | --- | --- | --- | --- | --- | --- | --- | --- | --- | --- | --- | --- | --- | --- | --- | --- | --- | --- | --- | --- | --- | --- | --- | --- | --- | --- | --- | --- | --- | --- | --- | --- | --- | --- | --- | --- | --- | --- | --- | --- | --- | --- | --- | --- | --- | --- | --- | --- | --- | --- | --- | --- | --- | --- | --- | --- | --- | --- | --- | --- | --- | --- | --- | --- | --- | --- | --- | --- | --- | --- | --- | --- | --- | --- | --- | --- | --- | --- | --- | --- | --- | --- | --- | --- | --- | --- | --- | --- | --- | --- | --- | --- | --- | --- | --- | --- | --- | --- | --- | --- | --- | --- | --- | --- | --- | --- | --- | --- | --- | --- | --- | --- | --- | --- | --- | --- | --- | --- | --- | --- | --- | --- | --- | --- | --- | --- | --- | --- | --- | --- | --- | --- | --- | --- | --- | --- | --- | --- | --- | --- | --- | --- | --- | --- | --- | --- | --- | --- | --- | --- | --- | --- | --- | --- | --- | --- | --- | --- | --- | --- | --- | --- | --- | --- | --- | --- | --- | --- | --- | --- | --- | --- | --- | --- | --- | --- | --- | --- | --- | --- | --- | --- | --- | --- | --- | --- | --- | --- | --- | --- | --- | --- | --- | --- | --- | --- | --- | --- | --- | --- | --- | --- | --- | --- | --- | --- | --- | --- | --- | --- | --- | --- | --- | --- | --- | --- | --- | --- | --- | --- | --- | --- | --- | --- | --- | --- | --- | --- | --- | --- | --- | --- | --- | --- | --- | --- | --- | --- | --- | --- | --- | --- | --- | --- | --- | --- | --- | --- | --- | --- | --- | --- | --- | --- | --- | --- | --- | --- | --- | --- | --- | --- | --- | --- | --- | --- | --- | --- | --- | --- | --- | --- | --- | --- | --- | --- | --- | --- | --- | --- | --- | --- | --- | --- | --- | --- | --- | --- | --- | --- | --- | --- | --- | --- | --- | --- | --- | --- | --- | --- | --- | --- | --- | --- | --- | --- | --- | --- | --- | --- | --- | --- | --- | --- | --- | --- | --- | --- | --- | --- | --- | --- | --- | --- | --- | --- | --- | --- | --- | --- | --- | --- | --- | --- | --- | --- | --- | --- | --- | --- | --- | --- | --- | --- | --- | --- | --- | --- | --- | --- | --- | --- | --- | --- | --- | --- | --- | --- | --- | --- | --- | --- | --- | --- | --- | --- | --- | --- | --- | --- | --- | --- | --- | --- | --- | --- | --- | --- | --- | --- | --- | --- | --- | --- | --- | --- | --- | --- | --- | --- | --- | --- | --- | --- | --- | --- | --- | --- | --- | --- | --- | --- | --- | --- | --- | --- | --- | --- | --- | --- | --- | --- | --- | --- | --- | --- | --- | --- | --- | --- | --- | --- | --- | --- | --- | --- | --- | --- | --- | --- | --- | --- | --- | --- | --- | --- | --- | --- | --- | --- | --- | --- | --- | --- | --- | --- | --- | --- | --- | --- | --- | --- | --- | --- | --- | --- | --- | --- | --- | --- | --- | --- | --- | --- | --- | --- | --- | --- | --- | --- | --- | --- | --- | --- | --- | --- | --- | --- | --- | --- | --- | --- | --- | --- | --- | --- | --- | --- | --- | --- | --- | --- | --- | --- | --- | --- | --- | --- | --- | --- | --- | --- | --- | --- | --- | --- | --- | --- | --- | --- | --- | --- | --- | --- | --- | --- | --- | --- | --- | --- | --- | --- | --- | --- | --- | --- | --- | --- | --- | --- | --- | --- | --- | --- | --- | --- | --- | --- | --- | --- | --- | --- | --- | --- | --- | --- | --- | --- | --- | --- | --- | --- | --- | --- | --- | --- | --- | --- | --- | --- | --- | --- | --- | --- | --- | --- | --- | --- | --- | --- | --- | --- | --- | --- | --- | --- | --- | --- | --- | --- | --- | --- | --- | --- | --- | --- | --- | --- | --- | --- | --- | --- | --- | --- | --- | --- | --- | --- | --- | --- | --- | --- | --- | --- | --- | --- | --- | --- | --- | --- | --- | --- | --- | --- | --- | --- | --- | --- | --- | --- | --- | --- | --- | --- | --- | --- | --- | --- | --- | --- | --- | --- | --- | --- | --- | --- | --- | --- | --- | --- | --- | --- | --- | --- | --- | --- | --- | --- | --- | --- | --- | --- | --- | --- | --- | --- | --- | --- | --- | --- | --- | --- | --- | --- | --- | --- | --- | --- | --- | --- | --- | --- | --- | --- | --- | --- | --- | --- | --- | --- | --- | --- | --- | --- | --- | --- | --- | --- | --- | --- | --- | --- | --- | --- | --- | --- | --- | --- | --- | --- | --- | --- | --- | --- | --- | --- | --- | --- | --- | --- | --- | --- | --- | --- | --- | --- | --- | --- | --- | --- | --- | --- | --- | --- | --- | --- | --- | --- | --- | --- | --- | --- | --- | --- | --- | --- | --- | --- | --- | --- | --- | --- | --- | --- | --- | --- | --- | --- | --- | --- | --- | --- | --- | --- | --- | --- | --- | --- | --- | --- | --- | --- | --- | --- | --- | --- | --- | --- | --- | --- | --- | --- | --- | --- | --- | --- | --- | --- | --- | --- | --- | --- | --- | --- | --- | --- | --- | --- | --- | --- | --- | --- | --- | --- | --- | --- | --- | --- | --- | --- | --- | --- | --- | --- | --- | --- | --- | --- | --- | --- | --- | --- | --- | --- | --- | --- | --- | --- | --- | --- | --- | --- | --- | --- | --- | --- | --- | --- | --- | --- | --- | --- | --- | --- | --- | --- | --- | --- | --- | --- | --- | --- | --- | --- | --- | --- | --- | --- | --- | --- | --- | --- | --- | --- | --- | --- | --- | --- | --- | --- | --- | --- | --- | --- | --- | --- | --- | --- | --- | --- | --- | --- | --- | --- | --- | --- | --- | --- | --- | --- | --- | --- | --- | --- | --- | --- | --- | --- | --- | --- | --- | --- | --- | --- | --- | --- | --- | --- | --- | --- | --- | --- | --- | --- | --- | --- | --- | --- | --- | --- | --- | --- | --- | --- | --- | --- | --- | --- | --- | --- | --- | --- | --- | --- | --- | --- | --- | --- | --- | --- | --- | --- | --- | --- | --- | --- | --- | --- | --- | --- | --- | --- | --- | --- | --- | --- | --- | --- | --- | --- | --- | --- | --- | --- | --- | --- | --- | --- | --- | --- | --- | --- | --- | --- | --- | --- | --- | --- | --- | --- | --- | --- | --- | --- | --- | --- | --- | --- | --- | --- | --- | --- | --- | --- | --- | --- | --- | --- | --- | --- | --- | --- | --- | --- | --- | --- | --- | --- | --- | --- | --- | --- | --- | --- | --- | --- | --- | --- | --- | --- | --- | --- | --- | --- | --- | --- | --- | --- | --- | --- | --- | --- | --- | --- | --- | --- | --- | --- | --- | --- | --- | --- | --- | --- | --- | --- | --- | --- | --- | --- | --- | --- | --- | --- | --- | --- | --- | --- | --- | --- | --- | --- | --- | --- | --- | --- | --- | --- | --- | --- | --- | --- | --- | --- | --- | --- | --- | --- | --- | --- | --- | --- | --- | --- | --- | --- | --- | --- | --- | --- | --- | --- | --- | --- | --- | --- | --- | --- | --- | --- | --- | --- | --- | --- | --- | --- | --- | --- | --- | --- | --- | --- | --- | --- | --- | --- | --- | --- | --- | --- | --- | --- | --- | --- | --- | --- | --- | --- | --- | --- | --- | --- | --- | --- | --- | --- | --- | --- | --- | --- | --- | --- | --- | --- | --- | --- | --- | --- | --- | --- | --- | --- | --- | --- | --- | --- | --- | --- | --- | --- | --- | --- | --- | --- | --- | --- | --- | --- | --- | --- | --- | --- | --- | --- | --- | --- | --- | --- | --- | --- | --- | --- | --- | --- | --- | --- | --- | --- | --- | --- | --- | --- | --- | --- | --- | --- | --- | --- | --- | --- | --- | --- | --- | --- | --- | --- | --- | --- | --- | --- | --- | --- | --- | --- | --- | --- | --- | --- | --- | --- | --- | --- | --- | --- | --- | --- | --- | --- | --- | --- | --- | --- | --- | --- | --- | --- | --- | --- | --- | --- | --- | --- | --- | --- | --- | --- | --- | --- | --- | --- | --- | --- | --- | --- | --- | --- | --- | --- | --- | --- | --- | --- | --- | --- | --- | --- | --- | --- | --- | --- | --- | --- | --- | --- | --- | --- | --- | --- | --- | --- | --- | --- | --- | --- | --- | --- | --- | --- | --- | --- | --- | --- | --- | --- | --- | --- | --- | --- | --- | --- | --- | --- | --- | --- | --- | --- | --- | --- | --- | --- | --- | --- | --- | --- | --- | --- | --- | --- | --- | --- | --- | --- | --- | --- | --- | --- | --- | --- | --- | --- | --- | --- | --- | --- | --- | --- | --- | --- | --- | --- | --- | --- | --- | --- | --- | --- | --- | --- | --- | --- | --- | --- | --- | --- | --- | --- | --- | --- | --- | --- | --- | --- | --- | --- | --- | --- | --- | --- | --- | --- | --- | --- | --- | --- | --- | --- | --- | --- | --- | --- | --- | --- | --- | --- | --- | --- | --- | --- | --- | --- | --- | --- | --- | --- | --- | --- | --- | --- | --- | --- | --- | --- | --- | --- | --- | --- | --- | --- | --- | --- | --- | --- | --- | --- | --- | --- | --- | --- | --- | --- | --- | --- | --- | --- | --- | --- | --- | --- | --- | --- | --- | --- | --- | --- | --- | --- | --- | --- | --- | --- | --- | --- | --- | --- | --- | --- | --- | --- | --- | --- | --- | --- | --- | --- | --- | --- | --- | --- | --- | --- | --- | --- | --- | --- | --- | --- | --- | --- | --- | --- | --- | --- | --- | --- | --- | --- | --- | --- | --- | --- | --- | --- | --- | --- | --- | --- | --- | --- | --- | --- | --- | --- | --- | --- | --- | --- | --- | --- | --- | --- | --- | --- | --- | --- | --- | --- | --- | --- | --- | --- | --- | --- | --- | --- | --- | --- | --- | --- | --- | --- | --- | --- | --- | --- | --- | --- | --- | --- | --- | --- | --- | --- | --- | --- | --- | --- | --- | --- | --- | --- | --- | --- | --- | --- | --- | --- | --- | --- | --- | --- | --- | --- | --- | --- | --- | --- | --- | --- | --- | --- | --- | --- | --- | --- | --- | --- | --- | --- | --- | --- | --- | --- | --- | --- | --- | --- | --- | --- | --- | --- | --- | --- | --- | --- | --- | --- | --- | --- | --- | --- | --- | --- | --- | --- | --- | --- | --- | --- | --- | --- | --- | --- | --- | --- | --- | --- | --- | --- | --- | --- | --- | --- | --- | --- | --- | --- | --- | --- | --- | --- | --- | --- | --- | --- | --- | --- | --- | --- | --- | --- | --- | --- | --- | --- | --- | --- | --- |
